# Molecular Basis of XRN2-Deficient Cancer Cell Sensitivity to Poly(ADP-ribose) Polymerase Inhibition

**DOI:** 10.3390/cancers16030595

**Published:** 2024-01-30

**Authors:** Talysa Viera, Quinn Abfalterer, Alyssa Neal, Richard Trujillo, Praveen L. Patidar

**Affiliations:** Department of Chemistry, New Mexico Institute of Mining and Technology, Socorro, NM 87801, USA

**Keywords:** XRN2, PARP1, R-loop, synthetic lethality, DSB repair, replication stress

## Abstract

**Simple Summary:**

Many cancers exhibit compromised 5′-3′-exoribonuclease 2 (XRN2) expression. XRN2 is a major regulator of RNA polymerase II (RNAPII) at the transcription termination sites of protein-coding genes. Deregulated transcription termination facilitates the formation of triple-stranded nucleic acid structures known as R-loops (RNA–DNA hybrids with displaced single-strand DNA). Elevated levels of unscheduled R-loops promote genomic instability. In the absence of XRN2, R-loop levels increase and promote DNA damage that activates DNA damage surveillance protein poly(ADP-ribose) polymerase 1 (PARP1). Previously, we discovered that the simultaneous absence of XRN2 and PARP1 compromises the survival of non-cancer and cancer cells; however, the underlying cellular stress response remained unknown. Here, we aimed to uncover the molecular consequences of concurrent XRN2 depletion and PARP1 inhibition. Our findings provide a mechanistic understanding of why cancer cells rely on PARP1 when XRN2 is absent and strengthen the translational aspect of targeting XRN2 cancer vulnerabilities using PARP inhibitors.

**Abstract:**

R-loops (RNA–DNA hybrids with displaced single-stranded DNA) have emerged as a potent source of DNA damage and genomic instability. The termination of defective RNA polymerase II (RNAPII) is one of the major sources of R-loop formation. 5′-3′-exoribonuclease 2 (XRN2) promotes genome-wide efficient RNAPII termination, and XRN2-deficient cells exhibit increased DNA damage emanating from elevated R-loops. Recently, we showed that DNA damage instigated by XRN2 depletion in human fibroblast cells resulted in enhanced poly(ADP-ribose) polymerase 1 (PARP1) activity. Additionally, we established a synthetic lethal relationship between XRN2 and PARP1. However, the underlying cellular stress response promoting this synthetic lethality remains elusive. Here, we delineate the molecular consequences leading to the synthetic lethality of XRN2-deficient cancer cells induced by PARP inhibition. We found that XRN2-deficient lung and breast cancer cells display sensitivity to two clinically relevant PARP inhibitors, Rucaparib and Olaparib. At a mechanistic level, PARP inhibition combined with XRN2 deficiency exacerbates R-loop and DNA double-strand break formation in cancer cells. Consistent with our previous findings using several different siRNAs, we also show that XRN2 deficiency in cancer cells hyperactivates PARP1. Furthermore, we observed enhanced replication stress in XRN2-deficient cancer cells treated with PARP inhibitors. Finally, the enhanced stress response instigated by compromised PARP1 catalytic function in XRN2-deficient cells activates caspase-3 to initiate cell death. Collectively, these findings provide mechanistic insights into the sensitivity of XRN2-deficient cancer cells to PARP inhibition and strengthen the underlying translational implications for targeted therapy.

## 1. Introduction

Safeguarding the integrity of the genome is a crucial cellular task. Compromised genomic integrity results in numerous devastating diseases such as neurodegenerative syndromes, autoimmune diseases, and cancer [1,2]. Triple-stranded nucleic structures containing RNA–DNA hybrids with displaced single-stranded DNA (R-loops) have emerged as a prominent source of DNA damage and genomic instability [3,4,5]. Inadequate regulation of RNA polymerase II (RNAPII) at termination sites of protein-coding genes promotes unscheduled R-loop formation. 5′-3′-exoribonuclease 2 (XRN2) is a major termination factor that cleaves RNAPII-associated RNA and promotes genome-wide poly(A) site-dependent RNAPII termination [6,7,8,9,10,11]. XRN2 is also involved in the coupled decapping of nascent transcripts and premature termination to confine bidirectional RNAPII elongation [12]. Moreover, XRN2 stimulates premature termination in a microprocessor-dependent manner and is involved in restricting the levels of promoter-associated non-productive transcripts [13,14]. Furthermore, XRN2 coordinates with p54(nrb) and PSF (Polypyrimidine Tract-Binding Protein-Associated Splicing Factor) to facilitate pre-mRNA 3′ processing and promotes co-transcriptional degradation of aberrant pre-mRNA [15,16]. Additionally, XRN2 is involved in the general degradation of RNA, gene silencing, and rRNA maturation [17,18,19]. Overall, XRN2′s role in RNA metabolism has been studied extensively; however, its function in other cellular processes remains poorly understood [6,7,8,9,10,11,12,13,14,15,16,17,18,19,20]. Importantly, genetic alternations of XRN2, such as mutations, mRNA expression, and copy number variations, are common in a wide range of cancers; XRN2 polymorphisms contribute to an increased risk of spontaneous lung cancer [20,21,22,23]. Finally, XRN2 was recently reported to play an important role in the invasiveness of certain cancers, and alteration in its expression correlates with poor survival of cancer patients [24]. Hence, understanding the role of XRN2 in processes beyond RNA metabolism and delineating the cellular consequences of XRN2 deficiency are imperative to gain mechanistic insights into its various cellular functions. 

Previously, XRN2 deficiency has been shown to create a complex phenotype that includes elevated R-loop formation and subsequently increased DNA double-strand breaks (DSBs), delayed DSB repair kinetics, chromosomal aberrations, increased sensitivity to DNA damaging agents, and replication stress [25]. Recently, we characterized the XRN2 interactome using a strategy that combined proteomics (tandem affinity purification-mass spectrometry, TAP-MS), bioinformatics, genetics, biochemical, and biological approaches and revealed molecular links that connect XRN2 to novel biological processes and pathways [20]. We found that the XRN2 associates with several proteins involved in cellular processes separate from RNA metabolism, and novel major pathways related to XRN2 include cell cycle control of chromosomal replication and DSB repair by NHEJ [20]. We and others have also found that cellular XRN2 deficiency created by several different si/shRNAs constructs results in elevated poly(ADP-ribose) polymerase 1 (PARP1) catalytic activity and a synthetic lethal relationship of these cells with PARP1 depletion/inhibition [20,26]. Another recent study also reported that post-translational modification of XRN2 is important for its ability to prevent R-loop-induced genomic instability in cancer cells [27]. Collectively, these findings highlight the translational implications of targeting XRN2 cancer vulnerabilities with PARP inhibitors (PARPi). However, the mechanistic basis of the synthetic lethal relationship between XRN2 and PARP1 remains elusive. Moreover, establishing the broader applicability of clinically relevant PARP inhibitors on XRN2-deficient cancer cells is warranted.

In the current study, we investigated the molecular consequences of XRN2 deficiency in lung and breast cancer cells in conjunction with PARP inhibition via Rucaparib and Olaparib to gain mechanistic insights into the synthetic lethality of XRN2 and PARP1. First, we evaluated the sensitivity of XRN2-deficient lung and breast cancer cells against these clinically relevant PARP inhibitors and then delineated the cellular consequences of simultaneous XRN2 deficiency and loss of PARP1 catalytic function. Collectively, our study provides mechanistic insights into why XRN2-deficient cells display sensitivity to PARPi and strengthens the notion of targeting XRN2 vulnerabilities in cancer via PARP inhibition. 

## 2. Materials and Methods

### 2.1. Chemicals and Reagents 

Rucaparib and Olaparib were purchased from Selleck Chemicals LLC (Houston, TX, USA). Camptothecin (CPT) was purchased from Alfa Aesar (Haverhill, MA, USA). Hoechst 33258 dye was purchased from Sigma-Aldrich (St. Louis, MO, USA). RNase H was purchased from New England BioLabs (Ipswich, MA, USA). Prolonged gold antifade mounting medium containing DAPI was purchased from Invitrogen (Eugene, OR, USA). 

### 2.2. Antibodies 

XRN2 (H-3), PARP1 (F-2), and 53BP1 (E-10) antibodies were purchased from Santa Cruz Biotechnology (Dallas, TX, USA). PAR (1075), DNA-RNA hybrid (S9.6), and anti-mouse IgG-HRP antibodies were purchased from EMD Millipore (Burlington, MA, USA). A second DNA-RNA hybrid (S9.6) antibody was purchased from Kerafast (Boston, MA, USA). Cleaved caspase-3 (D175) (5A1E) and nucleolin (D4C70) antibodies were purchased from Cell Signaling Technology (Danvers, MA, USA). The α-tubulin (T9026) antibody was purchased from Sigma-Aldrich (St. Louis, MO, USA). An additional PAR antibody (4335-MC) was purchased from Trevigen (Gaithersburg, MD, USA). The pRPA32 (S4/S8) IHC antibody was purchased from Bethyl Laboratories, Inc. (Montgomery, TX, USA). Alexa Fluor 488 anti-mouse IgG, Alexa Fluor 488 anti-rabbit IgG, Alexa Fluor 594 anti-mouse IgG, and Alexa Fluor 594 anti-rabbit IgG were purchased from Invitrogen (Eugene, OR, USA).

### 2.3. Tissue Culture 

Human A549 (lung carcinoma) and MDA-MB-231 (mammary adenocarcinoma) cells were obtained from ATCC. A549 cells were maintained in DMEM media (Lonza, Walkersville, MD, USA) supplemented with L-glutamine and 10% FBS (standard DMEM media). MDA-MB-231 cells were maintained in RPMI media (Lonza, Walkersville, MD, USA) supplemented with 2 mM L-glutamine and 10% FBS. All cells were kept at 37 °C with 5% CO_2_. Cells were routinely monitored to confirm the absence of mycoplasma contamination.

### 2.4. RNAi and Transfection

Non-target control siRNAs (siSCR) were purchased from Sigma-Aldrich (St. Louis, MO, USA). siXRN2 and siPARP1 were purchased from Santa Cruz Biotechnology (Dallas, TX, USA). Note that several different siRNAs against XRN2, including those that were used in this study, were validated previously by our laboratory and others [20,25,26].

The general procedure for transient knockdown experiments is described previously [20]. Briefly, for a typical transient transfection, cells were plated in 100 mm dishes (1 × 10^6^ cells/dish) and allowed to adhere overnight. OptiMEM, Lipofectamine 2000 RNAiMax, and the indicated siRNAs were used at 1 nM concentrations (or 2 nM siSCR in the case of a double knockdown). For immunofluorescent studies, the knockdowns were performed in 6-well plates after the cells had adhered to the glass coverslips. All experiments were performed within a 72 h knockdown window following transfection.

### 2.5. DNA Assay 

A modified cell survival assay measuring DNA content over 4-day period was utilized [28]. Following a 24 h transient siRNA transfection (1 nM siSCR or siXRN2), cells were seeded at 4000 cells/well in 96-well plates in 100 μL of media. The next day, media were aspirated and replaced with 100 μL of fresh media containing the indicated concentrations of Rucaparib (μM) or Olaparib (μM). The cells were exposed to PARPi for 24 h, and the media were again aspirated and replaced with fresh media (without PARPi). The cells were then allowed to grow until the control samples became confluent. The cells were then lysed in 50 μL water, freeze–thawed, and then treated with 100 μL 1X TNE buffer containing Hoechst 33258 fluorescence dye for 3 h at room temperature, and the DNA content was determined by measuring the fluorescence signal (355 nm/460 nm, 0.1 s) using a Victor X5 plate reader (PerkinElmer, Waltham, MA, USA). Fluorescence values of treated samples were normalized to the control DMSO samples and plotted as means ± SEM for treated over control (T/C) samples. The reported values are the result of *n* ≥ 4 biological repeats. 

### 2.6. Colony-Forming Assay 

A549 or MDA-MB-231 cells were seeded on 6-well plates at 250, 100, or 50 cells per well following transient siSCR or siXRN2 (1 nM) transfections for 24 h. The next day, cells were treated with 10 μM Rucaparib or 10 μM Olaparib for 24 h. Following treatment, the media were replaced with fresh media, and the cells were allowed to grow for 10 days. Then, the media was removed, and colonies were stained with crystal violet solution (1X PBS, 1% formaldehyde, 1% methanol, and 0.05% *w*/*v* crystal violet) for 20 min, thoroughly rinsed in water, and allowed to dry. Plates were imaged on an Azure c600 (Azure Biosystems, Dublin, CA, USA), and the colonies were counted using ImageJ. The counted colonies were normalized to the control, and data (means ± S.D.) were expressed as treated/control (T/C) samples. The reported values are the result of *n* ≥ 3 biological repeats. 

### 2.7. Western Blot

The standard Western blotting protocol was followed as previously described, with the indicated changes [20]. For a typical Western blotting experiment, 1 × 10^6^ cells were plated in 35 mm or 100 mm dishes and allowed to adhere overnight. The next day, cells were knocked down with the specified siRNA followed by the treatments and times as indicated. Cells were then lysed in ice-cold RIPA buffer (Alfa Aesar, Haverhill, MA, USA) containing 1X protease and 1X phosphatase inhibitors (Thermo Fisher Scientific, Waltham, MA, USA). Whole-cell protein extracts were then sonicated and centrifuged at 14,500 rpm at 4 °C, and the supernatants were obtained. Protein concentrations were determined via the BCA assay (Thermo Fisher Scientific). Next, 15 μg of protein were separated using SDS-PAGE gels and transferred to nitrocellulose or PVDF membranes. The membranes were blocked in either 1X casein blocking buffer (Sigma-Aldrich) or 5% skim milk-TBST for 1 h and incubated with primary antibodies overnight at 4 °C. Following washing, blots were incubated with appropriate secondary antibodies conjugated with HRP for 1 h at room temperature. Unless otherwise stated, primary antibodies were diluted at a concentration of 1:1000 in blocking buffer, α-tubulin was diluted at 1:5000, and all secondary IgG-HRP antibodies were diluted at 1:5000. Protein bands were detected using SuperSignal West Pico PLUS Chemiluminescent Substrate (Thermo Fisher Scientific) and imaged on an Azure c600 (Azure Biosystems). For quantification of Western blot images, protein band intensities were analyzed using ImageJ software (NIH; version 1.53c, http:imagej.net; accessed on 20 July 2020), and bands were normalized to the loading control. The reported relative intensities are the results of *n* ≥ 3. 

### 2.8. Immunofluorescence 

Immunofluorescence confocal microscopy was performed as previously described [29]. For a typical immunofluorescence experiment, cells were seeded on 6-well plates (75,000 cells/well) containing glass slides and allowed to adhere overnight. The next day, the cells were knocked down with 1 nM siRNA for 24 h. Cells were then treated with 10 μM Rucaparib for 12, 24, or 48 h or DMSO vehicle control. Depending on the experiment, either 25 μM Camptothecin (CPT) for 2 h or 1 mM H_2_O_2_ in 1X PBS for 15 min was used as a positive control. Following treatments, cells were washed with 1X PBS and fixed using ice-cold methanol/acetic acid (3:1, *v*/*v*) overnight at −20 °C. Fixed cells were then rehydrated in 1X PBS at room temperature (3x, 5 min each). Next, cells were then blocked in 1X PBS containing 5% normal goat serum for 1 h at room temperature. The cells were then incubated with the primary antibody in 1X PBS containing 5% normal goat serum for 3 h at room temperature. Cells were washed (3x, 5 min each in 1X PBS containing 0.05% Tween-20) and then incubated with the appropriate fluorescently tagged secondary antibody in 1X PBS containing 5% normal goat serum for 1 h at room temperature. The following antibody dilutions were used: anti-S9.6 (1:500), anti-nucleolin (1:2000), anti-53BP1 (1:750), anti-PAR (1:1000), anti-pRPA32 (1:1000), anti-cleaved caspase-3 (1:1500), and Alexa Fluor 488 or Alexa Fluor 594 (1:1000 or 1:1500). Finally, cells were washed (3x, 5 min each in 1X PBS containing 0.05% Tween-20), and the glass slides were mounted with prolong gold antifade mounting medium containing DAPI and sealed with nail polish. 

For enzymatic treatment involving RNase H, the general procedure was adopted from a previous study with the indicated changes [30]. Slides were rehydrated in PBS as stated above and subjected to blocking for 30 min in 5% goat serum. Next, slides were treated with 2.5 U of RNase H1 (New England BioLabs, Ipswich, MA, USA) supplemented with 3 mM magnesium chloride in 5% goat serum for 1 h at room temperature. The slides were then rinsed in blocking buffer for 5 min and followed by the standard IF protocol described above.

Images were acquired using the Olympus FV10i confocal laser scanning microscope (Olympus, Golden, CO, USA) with a 60x oil immersion objective using a 2.0 aperture. Laser and intensity settings were kept constant based on the positive control. The background was reduced using Olympus FluoView version 4.2b. Raw images were imported into ImageJ, and the nuclear foci were determined using the BioVoxxel plugin v2.5.1. For PAR and cleaved-caspase-3 IF, nuclear or cellular fluorescence intensities were then quantified and normalized to the siSCR DMSO negative control. The reported values are representative of *n* ≥ 3 biological repeats. 

### 2.9. Dot Blot Assay

For R-loop detection via dot blot assay, a previously described method was employed with indicated changes [31]. Cells were first lysed in TE buffer containing 0.5% SDS with 200 µg/mL of RNase A (Thermo Fisher Scientific) for 3 h at 37 °C, followed by the addition of 160 µg/mL proteinase K (New England BioLabs) and incubated at 37 °C overnight, phase-separated using phenol/chloroform/isoamyl alcohol (25:24:1), ethanol precipitated by adding 2 volumes of 100% ethanol and 100 µL of 7.5 M ammonium acetate overnight, washed 3 times with 70% ethanol, air dried, and resuspended in TE buffer. Genomic DNA was then fragmented by sonication and quantified. Genomic DNA samples were spotted on a nitrocellulose membrane, crosslinked with UV light (254 nm, 5 min), blocked with 1X Casein buffer at 4 °C for 1 h, and incubated with mouse S9.6 antibody (1:1000) overnight at 4 °C. After washing with TBS-Tween (0.1%), the membrane was incubated with HRP-conjugated anti-mouse secondary antibody (1:2000) at 4 °C for 3 h, followed by washing, and then developed. For RNase H treated control, genomic DNA was pre-incubated with 2.5 U of RNase H (New England BioLabs) for 3 h at 37 °C. For loading control, the membrane was stained using freshly made methylene blue staining solution (0.4 M sodium acetate, 0.4 M glacial acetic acid, 0.2% methylene blue) for 10 min and then briefly rinsed and imaged. For quantification, dot blot intensities were analyzed using ImageJ software (NIH; version 1.53c, http:imagej.net; accessed on 20 July 2020) and normalized to the siSCR or siSCR + RNase H negative control. The reported relative intensities are the results of *n* ≥ 3. 

### 2.10. Comet Assay 

The neutral comet assay was performed as described previously using the Comet Assay Kit (Trevigen, Gaithersburg, MD, USA) with the indicated changes [29]. A549 or MDA-MB-231 cells were plated on 6-well plates and adhered overnight. The next day, cells were knocked down with siSCR or siXRN2 (1 nM) for 24 h. Following transfection, the cells were treated with vehicle control (0.1% DMSO) or 10 μM Rucaparib for 48 h. A measure of 1 mM H_2_O_2_ in 1X PBS for 30 min was used as a positive control. Next, cells were trypsinized, washed, and resuspended in 1X PBS at a concentration of 2.5 × 10^5^ cells/mL, added to 37 °C LMAgarose (Trevigen) at a ratio of 1:10, and spread on a comet slide. The agarose was allowed to adhere to the slide at 4 °C for 30 min followed by overnight lysis at 4 °C. The next day, slides were submerged in 1X Neutral Electrophoresis Buffer for 30 min at 4 °C followed by electrophoresis at 20 V for 60 min at 4 °C in 1X Neutral Electrophoresis Buffer. Slides were placed in DNA precipitation solution (1 M ammonium acetate in 95% EtOH) for 30 min at room temperature, and then immersed in 70% EtOH for 30 min at room temperature. Next, the slides were dried at 37 °C for 10 min and stained with 1:10,000 dilution of SYBR Green in TE buffer (10 mM Tris-HCl pH 7.5 with 1 mM EDTA) for 30 min at room temperature. Slides were rinsed in distilled water and dried before imaging. The images were obtained using an Olympus FV10i confocal laser scanning microscope with a 10x objective. The comets were analyzed using the OpenComet v1.3 (www.biocomet.org; accessed on 10 November 2020) ImageJ (version 1.53c, http:imagej.net; accessed on 20 July 2020) plug-in. The minimum biological replicate size was *n* = 3.

### 2.11. Statistical Analyses 

Unless otherwise stated, the graphed data represent mean ± SEM. For the DNA content assays, two-tailed Student’s *t*-tests were performed using the Holm–Sidak method to correct for multiple comparisons. For all other data, an ordinary one-way ANOVA using the Dunnett’s multiple comparisons test was used to compare treated samples to control. The minimum biological replicate size was *n* = 3. Alpha was set to 0.05. GraphPad Prism 8 was used to perform the statistical analyses. * *p* < 0.05; ** *p* < 0.01, *** p < 0.001, **** *p* < 0.0001. 

## 3. Results

### 3.1. XRN2 Deficiency Sensitizes Cancer Cells to PARP Inhibition

**Figure 1:**
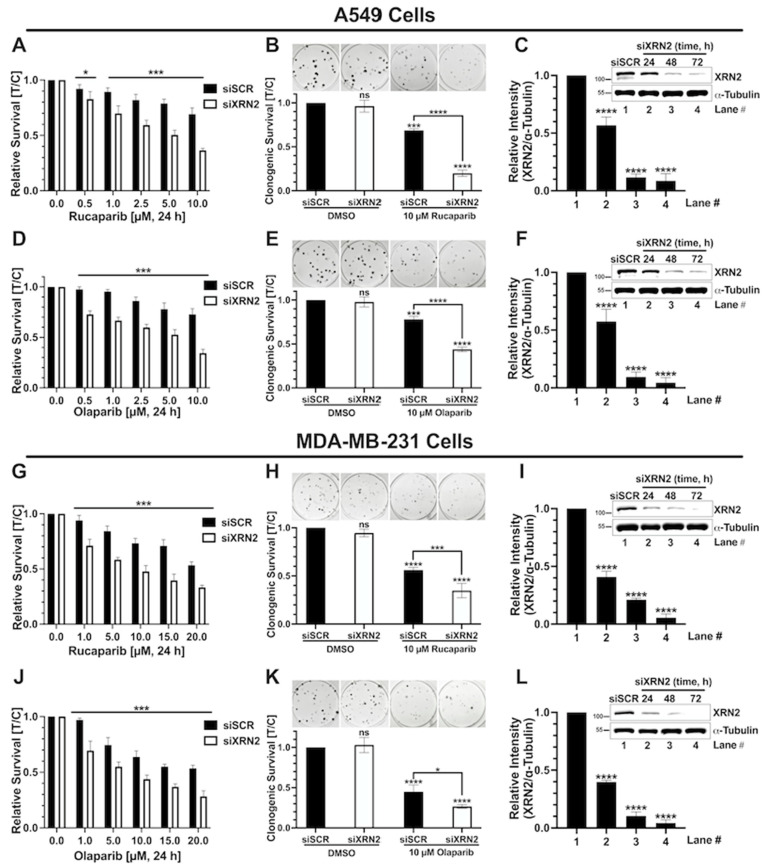
XRN2 knockdown sensitizes A549 and MDA-MB-231 cells to PARP1 inhibition. A549 (**A**–**F**) and MDA-MB-231 (**G**–**L**) cells treated with FDA-approved PARP1 inhibitors. (**A**) DNA assay of siSCR control (black bars) or siXRN2 (white bars) knockdown A549 cells treated with varying concentrations of Rucaparib for 24 h. (**B**) Colony-forming assay of A549 cells ±siXRN2 treated with 10 μM of Rucaparib or DMSO vehicle control. (**C**) Representative Western blot image of the siXRN2 knockdown confirmation and quantification with α-tubulin as a loading control. (**D**) DNA assay of A549 cells ±siXRN2 treated with varying concentrations of Olaparib for 24 h. (**E**) Colony-forming assay of A549 cells ±siXRN2 treated with 10 μM of Olaparib or DMSO vehicle control. (**F**) Representative Western blot image of the siXRN2 knockdown confirmation and quantification with α-tubulin as a loading control. (**G**) DNA assay of siSCR control or siXRN2 knockdown MDA-MB-231 cells treated with varying concentrations of Rucaparib for 24 h. (**H**) Colony-forming assay of MDA-MB-231 cells ±siXRN2 treated with 10 μM of Rucaparib or DMSO vehicle control. (**I**) Representative Western blot image of the siXRN2 knockdown confirmation and quantification with α-tubulin as a loading control. (**J**) DNA assay of MDA-MB-231 cells ±siXRN2 treated with varying concentrations of Olaparib for 24 h. (**K**) Colony-forming assay of MDA-MB-231 cells ±siXRN2 treated with 10 μM of Olaparib or DMSO vehicle control. (**L**) Representative Western blot image of the siXRN2 knockdown confirmation and quantification with α-tubulin as a loading control. For the DNA content assays, *p*-values were obtained via two-tailed students tests. *, *p* < 0.05; ***, *p* < 0.001. For the clonogenic survival assays and Western blot comparisons, *p*-values were obtained via an ordinary one-way ANOVA using the Dunnett’s multiple comparisons test. ***, *p* < 0.001; ****, *p* < 0.0001; ns, not significant. The uncropped blots are shown in Supplementary Figure S1.

Efficient transcription termination facilitated by XRN2 aids in R-loop resolution and when it is deficient in cells, R-loops have been shown to accumulate [25,26,32]. As a consequence of defective termination promoting R-loops, XRN2-deficient cells show elevated DNA damage and PARP1 hyperactivation [20,25,26]. In our recent study, a genetic approach led us to demonstrate the synthetic lethal relationship between XRN2 and PARP1 depletion, and we also found that XRN2 depletion in immortalized human fibroblast cells elicits sensitivity to PARP inhibitor (BMN 673 (Talazoparib)) treatment [25]. To ensure the broad applicability of PARP inhibitors (PARPi) against XRN2 vulnerabilities, here, we evaluated the effects that other FDA-approved PARPi, Rucaparib, and Olaparib have in XRN2-depleted A549 (lung carcinoma) and MDA-MB-231 (mammary adenocarcinoma) cells (Figure 1). Transient depletion of XRN2 in the current study was achieved using siRNAs that were validated in our previous study, effectively eliminating the issues related to off-target effects of siRNAs [20,25]. The concentrations of Rucaparib and Olaparib tested were based on reported IC_50_ values against a panel of NCI60 cell lines and other previously reported values [33,34,35]. Cell survival was evaluated via the DNA assay and clonogenic survival assay, as described in detail under Section 2, “Materials and Methods”. Rucaparib significantly decreased the cell survival of A549 XRN2 knockdown cells (siXRN2) at multiple concentrations compared to control knockdown (siSCR) cells measured via the DNA content assay (Figure 1A). Additionally, A549 siXRN2 cells treated with Rucaparib (10 μM, 24 h) showed significantly reduced clonogenic survival compared to siSCR cells (Figure 1B). Successful XRN2 knockdown within the treatment window pertinent to Figure 1A,B was validated through Western blot (Figure 1C). Similar cell survival trends were observed in A549 siXRN2 cells treated with Olaparib when compared to the siSCR control (Figure 1D–F). Importantly, we then assessed the effect XRN2-depletion has in combination with PARPi in the triple-negative breast cancer cell line, MDA-MB-231 (Figure 1G–L). Similar to the A549 lung cancer cells, we observed a significant decrease in cell survival in MDA-MB-231 siXRN2 cells treated with PARPi when compared to the siSCR control for Rucaparib (Figure 1G–I) and Olaparib (Figure 1J–L). We treated these cells with up to 20 μM PARPi based on previously reported IC_50_ values [35]. Of note, despite ~95% knockdown efficiency at 1 nM siRNA, XRN2 depletion alone did not compromise cell survival, as highlighted in the siXRN2 DMSO control in both cell lines. Collectively, these data clearly show the potential of various FDA-approved PARPi to sensitize XRN2-depleted cancer cells. 

### 3.2. Simultaneous XRN2 Depletion and PARP Inhibition Enhance R-loop Formation 

**Figure 2:**
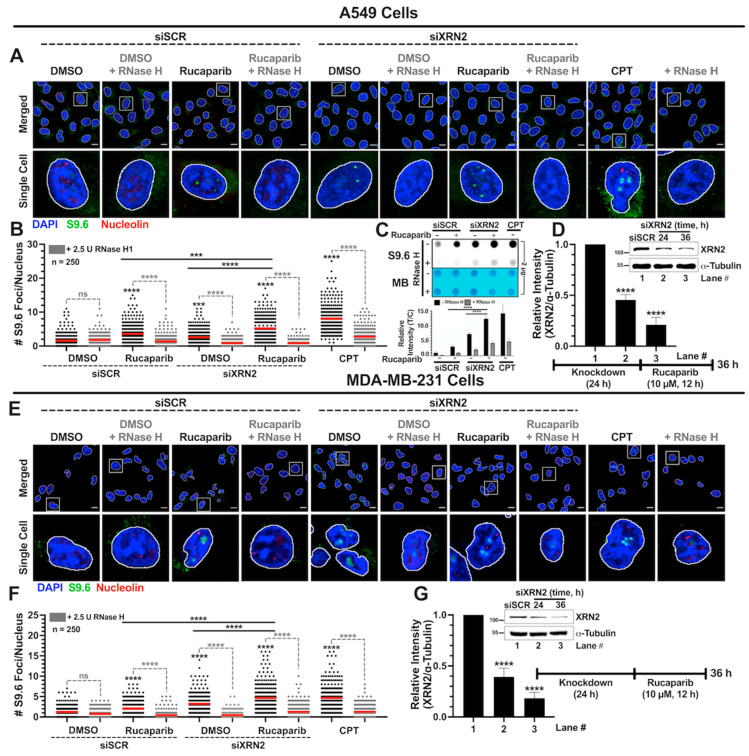
XRN2 depletion with simultaneous PARP1 inhibition further enhances R-loop formation in cancer cells. (**A**) Representative confocal immunofluorescent (IF) microscopy images of nuclei stained with DAPI (blue), anti-S9.6 (green), and nucleolin (red) in A549 cells treated with ±siXRN2, ±10 μM Rucaparib, and ± 2.5 U RNase H. Cells treated with 25 μM CPT for 2 h served as a positive control. The scale bar is 10 μm. (**B**) Following subtraction of the nucleolin signal using ImageJ (version 1.53c, http://imagej.net; accessed on 20 July 2020), nuclear S9.6 foci were determined via the BioVoxxel ImageJ plugin v2.5.1 (www.biovoxxel.de; accessed on 21 January 2024). The quantification plot is representative of 3 biological replicates and indicates the number of nuclear foci in 250 individual cells. The red bar on each dataset represents the mean. (**C**) Representative dot blot image (top) and quantification (bottom) of A549 cells treated with ±siXRN2, ±10 μM Rucaparib, and ± 2.5 U RNase H. Cells treated with 25 μM CPT for 2 h served as a positive control. Dot blot stained with methylene blue (MB) was used as a loading control. The quantification plot is representative of 3 biological replicates. (**D**) Representative Western blot image and quantification of the siXRN2 knockdown confirmation with α-tubulin as a loading control. (**E**) Representative confocal immunofluorescent (IF) microscopy images of nuclei stained with DAPI (blue), anti-S9.6 (green), and nucleolin (red) in MDA-MB-231 cells treated with ±siXRN2, ±10 μM Rucaparib, and ±2.5 U RNase H. Cells treated with 25 μM CPT for 2 h served as a positive control. The scale bar is 10 μm. (**F**) Following subtraction of the nucleolin signal using ImageJ, nuclear S9.6 foci were determined. Light gray plots indicate treatment with RNase H. The quantification plot is representative of 3 biological replicates and indicates the number of nuclear foci in 250 individual cells. The red bar on each dataset represents the mean. (**G**) Representative Western blot image and quantification of the siXRN2 knockdown confirmation with α-tubulin as a loading control. The representative images show the whole cells with the nuclei outlined. The white box outlines the highlighted single cell. For the IF, dot blot, and Western blot analyses, *p*-values were obtained via an ordinary one-way ANOVA using the Dunnett’s multiple comparisons test. ***, *p* < 0.001; ****, *p* < 0.0001, comparing treatments to the control (siSCR + DMSO or + RNase H) or as indicated. The uncropped blots are shown in Supplementary Figure S2.

Similar to XRN2 depletion, PARP1 knockdown also results in elevated levels of R-loop formation [36]. It is conceivable that PARP1 inhibition may exert a similar effect as PARP1 knockdown in enhancing R-loop formation. Thus, it is important to evaluate the effect of XRN2 depletion in combination with PARP1 inhibition on R-loop formation to delineate the mechanistic basis of their synthetic lethal relationship. To address this, we utilized the effective conditions of PARP1 inhibition by Rucaparib defined in Figure 1 for all the studies described below. Nuclear R-loop foci were evaluated using the S9.6 antibody via immunofluorescence confocal microscopy as described in detail under Section 2. As a positive control, cells were treated with 25 μM camptothecin (CPT) for 2 h. Importantly, non-specific binding of S9.6 antibody is recognized as a major limitation of detecting genuine R-loop via immunofluorescence-based method [30]. To address this issue and to avoid S9.6 artifacts, several measures were considered in the present study that include quantifying nuclear R-loop foci, excluding S9.6 signal overlapping with nucleolin, and demonstrating RNase H sensitivity of the nuclear S9.6 signal. A549 cells depleted in XRN2 and treated with 10 μM Rucaparib displayed significantly higher R-loop formation when compared to the siSCR DMSO vehicle control, siSCR cells treated with 10 μM Rucaparib, and siXRN2 cells treated with DMSO (Figure 2A,B). Representative images shown in Figure 2 are whole-cell images with the nuclei outlined. Note that nuclear R-loop foci presented here are not overlapping with nucleolin and show clear sensitivity to RNase H treatment, indicating the genuine detection of R-loops in all of our samples. To further strengthen these findings, we performed dot blot analyses to detect R-loops in A549 cells using similar treatment conditions as described above. Dot blot analyses also clearly demonstrate that XRN2-deficient A549 cells treated with Rucaparib accumulate considerably higher RNase H-sensitive R-loops compared to the control (siSCR + DMSO) and individual (siSCR + Rucaparib or siXRN2 + DMSO) treatments (Figure 2C). Representative Western blot image along with the quantification, show that A549 cells used for Figure 2A–C were indeed depleted in XRN2 (Figure 2D). Consistent with A549 cells, MDA-MB-231 cells depleted in XRN2 and treated with 10 μM Rucaparib also exhibited significantly higher R-loop formation when compared to the siSCR cells with DMSO, siSCR cells treated with 10 μM Rucaparib, and XRN2-depleted cells with DMSO (Figure 2E–G). Together, these data suggest that the simultaneous depletion of XRN2 and pharmacological inhibition of PARP1 significantly elevate R-loop formation and likely contribute to the higher cellular sensitivity of the combination treatment. 

### 3.3. Concurrent XRN2 Depletion and PARP Inhibition Exacerbate DSB Formation and Downstream Signaling

**Figure 3:**
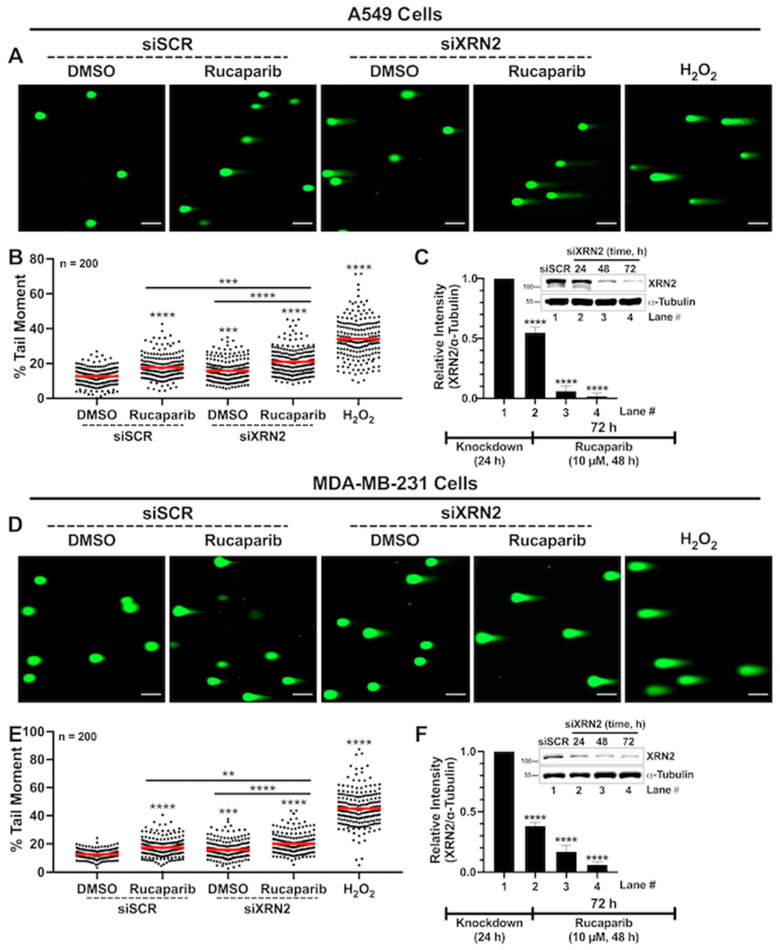
XRN2 depletion with simultaneous PARP1 inhibition elevates DSB formation in cancer cells. (**A**) Representative images of comets stained with SYBR Green in ±siXRN2 ±10 μM Rucaparib A549 cells. Cells treated with 1 mM H_2_O_2_ for 30 min served as a positive control. The scale bars are 100 μm. (**B**) Quantification of the comet tail moments from images processed in ImageJ (version 1.53c, http://imagej.net; accessed on 20 July 2020) utilizing the plugin OpenComet v1.3 (www.biocomet.org; accessed on 21 January 2024). The graph represents three biological repeats and describes the tail moments of 200 individual comets. The red bar on each dataset represents the mean. (**C**) Representative Western blot image and quantification indicating the successful knockdown of XRN2 in A549 cells with α-tubulin as a loading control. (**D**) Representative images of comets stained with SYBR Green in ±siXRN2 ±10 μM Rucaparib MDA-MB-231 cells. Cells treated with 1 mM H_2_O_2_ for 30 min served as a positive control. The scale bars are 100 μm. (**E**) Quantification of the comet tail moments from images processed using the OpenComet Image J plugin, as stated above. The graph represents three biological repeats and describes the tail moments of 200 individual comets. The red bar on each dataset represents the mean. (**F**) Representative Western blot image and quantification indicating the successful knockdown of XRN2 in MDA-MB-231 cells with α-tubulin as a loading control. *p*-values were obtained via an ordinary one-way ANOVA using the Dunnett’s multiple comparisons test. ** *p* < 0.01, ***, *p* < 0.001; ****, *p* < 0.0001, comparing treatments to the control (siSCR + DMSO) or as indicated. The uncropped blots are shown in Supplementary Figure S3.

**Figure 4:**
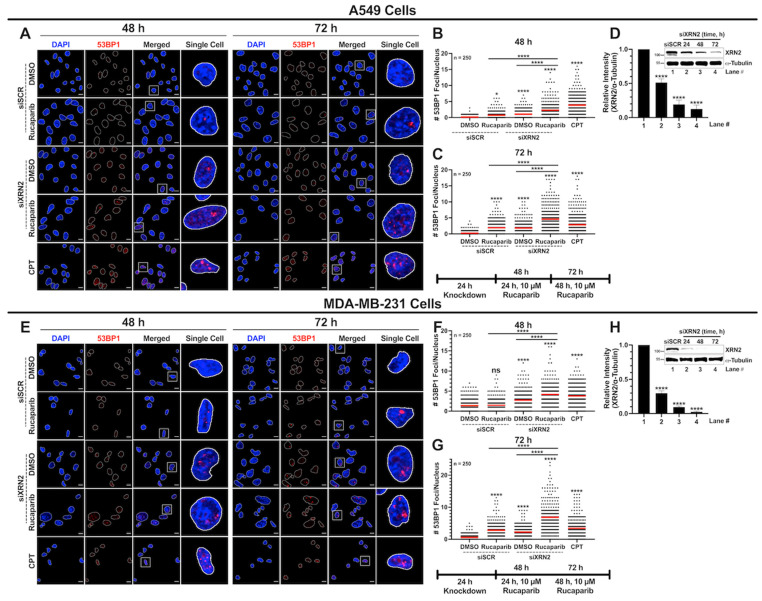
XRN2 depletion with simultaneous PARP1 inhibition increases DSB signaling in cancer cells. (**A**) Representative confocal immunofluorescence microscopy images of nuclei stained with DAPI (blue) and 53BP1 (red) in ±siXRN2 ±10 μM Rucaparib A549 cells. Cells treated with 25 μM CPT for 2 h served as a positive control. The scale bar is 10 μm. (**B**,**C**) Quantification of 53BP1 foci from images obtained from 48 h (**B**) and 72 h (**C**) processed in ImageJ. The graph is representative of 3 biological repeats and indicates the nuclear foci of 250 individual cells. The red bar on each dataset represents the mean. (**D**) Representative Western blot image and quantification indicating the successful knockdown of XRN2 in A549 cells with α-tubulin as a loading control. (**E**) Representative confocal immunofluorescence microscopy images of nuclei stained with DAPI (blue) and 53BP1 (red) in ±siXRN2 ±10 μM Rucaparib MDA-MB-231 cells. Cells treated with 25 μM CPT for 2 h served as a positive control. The scale bar is 10 μm. (**F**,**G**) Quantification of 53BP1 foci from images obtained from 48 h (**F**) and 72 h (**G**) processed in ImageJ. The graph is representative of 3 biological repeats and indicates the nuclear foci of 250 individual cells. The red bar on each dataset represents the mean. (**H**) Representative Western blot image and quantification indicating the successful knockdown of XRN2 in MDA-MB-231 cells with α-tubulin as a loading control. White boxes indicate highlighted Single Cell images. *p*-values were obtained via an ordinary one-way ANOVA using the Dunnett’s multiple comparisons test. * *p* < 0.05, ****, *p* < 0.0001, ns, not significant, comparing treatments to the control (siSCR + DMSO) or as indicated. The uncropped blots are shown in Supplementary Figure S4.

The increase in R-loop formation in cells simultaneously depleted in XRN2 and treated with PARP inhibitors prompted us to evaluate DNA double-strand break (DSB) formation as a contributing factor in enhancing cellular stress and promoting cell death (Figure 3 and Figure 4). To evaluate DSBs, we first utilized the neutral comet assay, as described in Section 2. A549 cells depleted in XRN2 and treated with Rucaparib showed significantly higher mean comet tail moments than cells treated with siSCR DMSO control, siSCR with 10 μM Rucaparib, or siXRN2 DMSO (Figure 3A–C). Similarly, MDA-MB-231 cells depleted in XRN2 and treated with 10 μM Rucaparib exhibited a significantly higher mean comet tail moment than cells treated with the siSCR DMSO control, siSCR with 10 μM Rucaparib, or siXRN2 DMSO (Figure 3D–F). H_2_O_2_ (1 mM for 30 min) served as a positive control for induction of DNA damage and displayed a strong comet tail moment for both A549 and MDA-MB-231 experiments (Figure 3A,B,D,E, respectively). 

To further support the elevated levels of DSBs instigated by XRN2 deficiency in conjunction with PARPi, we utilized immunofluorescence confocal microscopy to monitor 53BP1 foci as a marker of DSB signaling (Figure 4). Consistent with the comet assay data, A549 cells depleted in XRN2 and treated with PARPi demonstrated increased 53BP1 foci when compared to the siSCR DMSO control, siSCR with PARPi, and siXRN2 DMSO at both the 48 and 72 h time points (Figure 4A–C). Analogous to A549 cells, MDA-MB-231 cells depleted in XRN2 and treated with PARPi showed elevated 53BP1 foci compared to the siSCR DMSO control, siSCR with PARPi, and siXRN2 DMSO at both the 48 and 72 h time points (Figure 4E–G). CPT (25 µM for 2 h) was used as a positive control for both A549 and MDA-MB-231 experiments and displayed an expected increase in 53BP1 foci (Figure 4A–C,E–G, respectively). XRN2 depletion for the treatment window is confirmed for both A549 and MDA-MB-231 cells (Figure 4D,H, respectively). Taken together, data presented in Figure 3 and Figure 4 for A549 and MDA-MB-231 cancer cells clearly demonstrate that the concurrent depletion of XRN2 and PARPi exacerbate DSB formation at higher levels than each individual treatment alone and amplify the cellular stress response. 

### 3.4. XRN2 Deficiency Enhances PARP1 Activity in Cancer Cells

**Figure 5:**
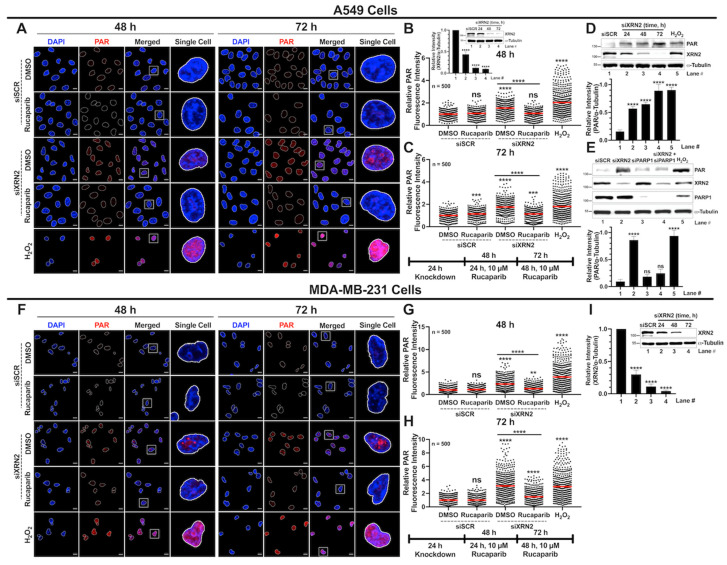
XRN2 depletion enhances PARP1 activity in cancer cells. (**A**) Representative confocal immunofluorescence microscopy images of nuclei stained with DAPI (blue) and PAR (red) in ±siXRN2 ±10 μM Rucaparib A549 cells. Cells treated with 1 mM H_2_O_2_ for 15 min served as a positive control. The scale bar is 10 μm. (**B**,**C**) Quantification of the nuclear PAR fluorescence signal from images obtained from 48 h (**B**) and 72 h (**C**) processed in ImageJ. The graph represents 3 biological repeats and indicates the nuclear PAR fluorescence intensities of 500 individual cells normalized to siSCR + DMSO. The red bar on each dataset represents the mean. (**B**, inset) Representative Western blot image and quantification indicating the successful knockdown of XRN2 in cells used in (**A**–**C**). (**D**) Representative Western blot image and quantification of PAR formation over time in A549 cells depleted in XRN2 for 24, 48, and 72 h with H_2_O_2_ (1 mM, 15 min) as a positive control with α-tubulin as a loading control. (**E**) Representative Western blot image and quantification of PAR formation in siSCR, siXRN2, siPARP1, and siXRN2 + siPARP1 A549 cells. The cells were transiently knocked down for 48 h. Also shown are the XRN2 and PARP1 knockdown confirmations with α-tubulin as a loading control. (**F**) Representative confocal immunofluorescence microscopy images of nuclei stained with DAPI (blue) and PAR (red) in ±siXRN2 ±10 μM Rucaparib MDA-MB-231 cells. Cells treated with 1 mM H_2_O_2_ for 15 min served as a positive control. The scale bar is 10 μm. (**G**,**H**) Quantification of the nuclear PAR fluorescence signal from images obtained from 48 h (**G**) and 72 h (**H**) processed in ImageJ. The graph represents 3 biological repeats and indicates the nuclear PAR fluorescence intensities of 500 individual cells normalized to siSCR + DMSO. The red bar on each dataset represents the mean. (**I**) Representative Western blot image and quantification indicating the successful knockdown of XRN2 in MDA-MB-231 cells with α-tubulin as a loading control. White squares indicate highlighted Single Cell. *p*-values were obtained via an ordinary one-way ANOVA using the Dunnett’s multiple comparisons test. ** *p* < 0.01, ***, *p* < 0.001; ****, *p* < 0.0001; ns, not significant, comparing treatments to the siSCR + DMSO control or as indicated. The uncropped blots are shown in Supplementary Figure S5.

We recently showed that immortalized human fibroblast cells deficient in XRN2 displayed increased PARP1 activity to counteract the DNA damage response and promote cell survival [20]. To further substantiate these findings, we sought to evaluate the effect that XRN2 depletion has on PARP1 activation in A549 and MDA-MB-231 cancer cells. We investigated PARP1 activity by measuring PAR (poly(ADP-ribose)) levels via immunofluorescence confocal microscopy and Western blot analysis as described in Section 2. A549 cells showed significantly higher PAR formation after XRN2 knockdown compared to the basal PAR levels of siSCR DMSO control at both the 48 and 72 h time points (Figure 5A–C). Moreover, Rucaparib treatment reduced the PAR levels of XRN2-deficient cells to that of siSCR control cells at 48 h (Figure 5B) and a significant reduction at 72 h (Figure 5C). H_2_O_2_-treated cells were used as positive control and showed strong PAR staining (Figure 5A–C). To further support PARP1 hyperactivation prompted by XRN2 deficiency, we utilized Western blot analysis to detect the increase in PAR formation in A549 cells. As XRN2 is depleted in A549 cells over time (24, 48, and 72 h), there is a significant increase in PAR formation when compared to the siSCR control knockdown (Figure 5D). Additionally, to emphasize the engagement of PARP1 in the absence of XRN2, we utilized a genetic approach and evaluated the effect of a double knockdown of XRN2 and PARP1 on PAR formation. A549 cells depleted in XRN2 hyperactivate PARP1, which is ameliorated with PARP1 depletion (Figure 5E). These findings are consistent with our immunofluorescence studies conducted with pharmacological inhibition of PARP1. Similar to A549 cells, MDA-MB-231 cells depleted in XRN2 showed a strong increase in PARP1 catalytic activity at both 48 and 72 h time points after knockdown and Rucaparib significantly blocked the formation of PAR (Figure 5F–I). Collectively, these data support the notion that cellular stress in the form of R-loops and DSBs induced by XRN2 depletion consequently hyperactivates PARP1. 

### 3.5. XRN2 Deficiency Combined with PARP Inhibition Results in Enhanced Replication Stress 

**Figure 6:**
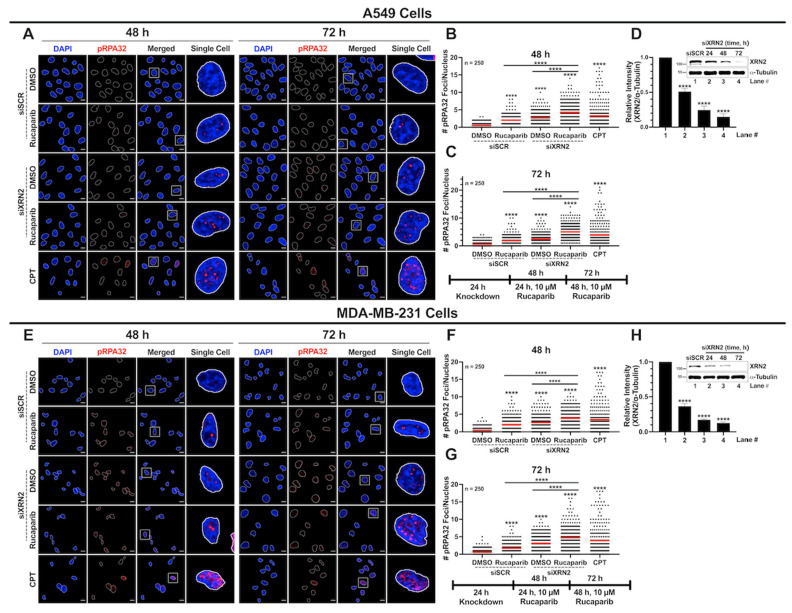
XRN2 depletion with simultaneous PARP1 inhibition increases replication stress in cancer cells. (**A**) Representative confocal immunofluorescence microscopy images of nuclei stained with DAPI (blue) and pRPA32 (red) in ±siXRN2 ±10 μM Rucaparib A549 cells. Cells treated with 25 μM CPT for 2 h served as a positive control. The scale bar is 10 μm. (**B**,**C**) Quantification of the nuclear foci from images obtained from 48 h (**B**) and 72 h (**C**) processed in ImageJ. The graph is representative of 3 biological repeats and indicates the nuclear pRPA32 foci in 250 cells. The red bar on each dataset represents the mean. (**D**) Representative Western blot image and quantification indicating the successful knockdown of XRN2 in A549 cells with α-tubulin as a loading control. (**E**) Representative confocal immunofluorescence microscopy images of nuclei stained with DAPI (blue) and pRPA32 (red) in ±siXRN2 ±10 μM Rucaparib A549 cells. Cells treated with 25 μM CPT for 2 h served as a positive control. The scale bar is 10 μm. (**F**,**G**) Quantification of the nuclear foci from images obtained from 48 h (**F**) and 72 h (**G**) processed in ImageJ. The graph is representative of 3 biological repeats and indicates the nuclear pRPA32 foci in 250 cells. (**H**) Representative Western blot image and quantification indicating the successful knockdown of XRN2 in A549 cells with α-tubulin as a loading control. White squares indicate highlighted Single Cell. *p*-values were obtained via an ordinary one-way ANOVA using the Dunnett’s multiple comparisons test. ****, *p* < 0.0001, comparing treatments to the control (siSCR + DMSO) or as indicated. The uncropped blots are shown in Supplementary Figure S6.

Replication stress is one of the major contributors inducing genomic instability, and conflicts between transcription and replication are a significant culprit [37]. Unresolved co-transcriptional R-loops have been shown to stall replication fork progression, replication fork collapse, and lead to toxic DSBs [38,39]. Thus, it is conceivable that enhanced replication stress driven by XRN2 deficiency combined with impaired PARP1 function could be a contributing factor in the synthetic lethality of XRN2 knockdown cells with PARPi. To test this notion, we sought to investigate the replication stress in XRN2-depleted cells treated with PARP1 inhibition by measuring phospho-RPA32(S4/S8) levels using immunofluorescence confocal microscopy as described in Section 2. A549 cells deficient in XRN2 and treated with Rucaparib demonstrated a significant increase in pRPA32(S4/S8) foci compared to the siSCR DMSO control, siSCR Rucaparib, and siXRN2 DMSO at both the 48 and 72 h time points (Figure 6A–D). Similar trends in pRPA32(S4/S8) foci formation were observed in MDA-MB-231 cells depleted in XRN2 and treated with 10 μM Rucaparib compared to the siSCR DMSO control at both the 48 and 72 h time points (Figure 6E–H). CPT-treated cells (25 µM for 2 h) were used as a positive control (Figure 6A–C,E–G). Collectively, these data demonstrate that enhanced replication stress is also an underlying cause of the synthetic lethality of XRN2 knockdown cells treated with PARPi.

### 3.6. Combined XRN2 Depletion and PARP Inhibition Activate Caspase-3

**Figure 7:**
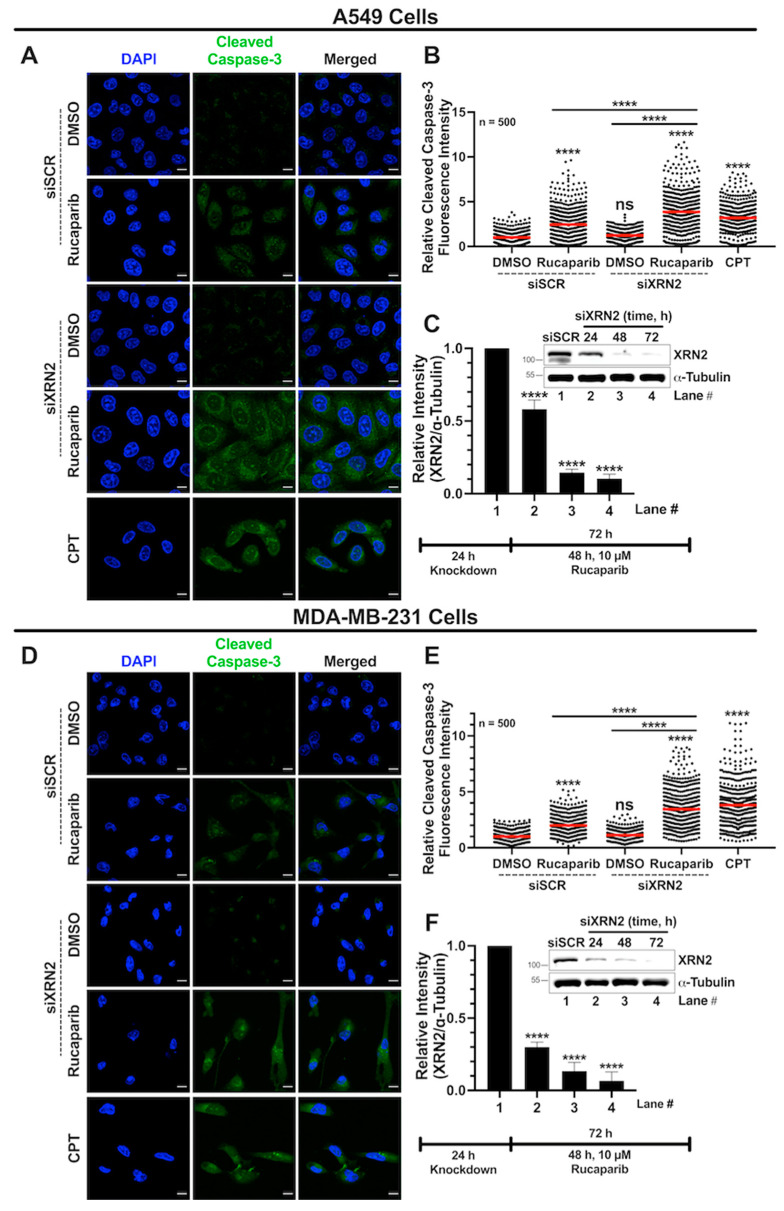
XRN2 depletion with PARP1 inhibition activates caspase-3 in cancer cells. (**A**) Representative confocal microscopy images evaluating the activation of caspase-3 using the cleaved caspase-3 antibody (green) and nuclei stained with DAPI (blue) in ±siXRN2 ±10 μM Rucaparib A549 cells. Cells treated with 10 μM CPT for 48 h were used as a positive control. The scale bar is 10 μm. (**B**) Quantification of the cleaved caspase-3 fluorescence signal from images processed in ImageJ. The graph represents three biological repeats and indicates the fluorescence intensities of 500 individual cells normalized to siSCR + DMSO. The red bar on each dataset represents the mean. (**C**) Representative Western blot image and quantification indicating the successful knockdown of XRN2 in A549 cells with α-tubulin as a loading control. (**D**) Representative confocal microscopy images evaluating the activation of caspase-3 using the cleaved caspase-3 antibody (green) and nuclei stained with DAPI (blue) in ±siXRN2 ±10 μM Rucaparib MDA-MB-231 cells. Cells treated with 10 μM CPT for 48 h were used as a positive control. The scale bar is 10 μm. (**E**) Quantification of the cleaved caspase-3 fluorescence signal from images processed in ImageJ. The graph represents three biological repeats and indicates the fluorescence intensities of 500 individual cells normalized to siSCR + DMSO. The red bar on each dataset represents the mean. (**F**) Representative Western blot image and quantification indicating the successful knockdown of XRN2 in MDA-MB-231 cells with α-tubulin as a loading control. *p*-values were obtained via an ordinary one-way ANOVA using the Dunnett’s multiple comparisons test. ****, *p* < 0.0001; ns, not significant, comparing treatments to the control (siSCR + DMSO) or as indicated. The uncropped blots are shown in Supplementary Figure S7.

After evaluating the synthetic lethality and underlying cellular stress responses in cancer cells with XRN2 depletion and PARP1 inhibition (Figure 1, Figure 2, Figure 3, Figure 4, Figure 5 and Figure 6), we investigated the cell death pathway employed under these conditions. We measured activated caspase-3 (cleaved caspase-3) levels via immunofluorescence confocal microscopy as described in Section 2. A549 cells deficient in XRN2 and treated with 10 μM Rucaparib exhibited a strong increase in activated caspase-3 levels compared to the siSCR DMSO control, siSCR with Rucaparib treatment, and siXRN2 (Figure 7A–C). XRN2-deficient MDA-MB-231 cells treated with 10 μM Rucaparib also showed significantly increased levels of cleaved caspase-3 compared to the siSCR DMSO control, siSCR with Rucaparib treatment, and siXRN2 (Figure 7D–F). CPT-treated cells (10 µM for 48 h) were used as a positive control (Figure 7A,B,D,E). Importantly, siXRN2 with DMSO alone did not cause a significant increase in the activated caspase-3 levels in either cell line. Together, these data indicate that the concurrent depletion of XRN2 and PARP inhibition activate caspase-3 to initiate cell death.

## 4. Discussion

Numerous studies have emphasized the significance of 5′-3′-exoribonuclease 2 (XRN2) and its homologs in RNA metabolism [6,7,8,12,13,14,15,16,17,18,19,20,25,26,32,40,41,42,43,44]. However, its function in genome maintenance has just begun to emerge [20,25,26,45,46]. In our previous study, we characterized the interactome of XRN2, where we reported the following: (i) XRN2 interacts with central DNA damage sensing protein poly(ADP-ribose) polymerase 1 (PARP1); (ii) XRN2-deficient fibroblast cells hyperactive PARP1 to alleviate cellular stress; (iii) XRN2-deficient fibroblast cells also show synthetic lethality with PARP1 depletion/inhibition. Here, we delineate the cellular consequences of XRN2 deficiency in cancer cells treated with clinically relevant PARP inhibitors (PARPi) to provide mechanistic insights into the synthetic lethal relationship between XRN2 and PARP1. The primary conclusion of this study is that, at the molecular level, the synthetic lethality of XRN2 and PARP1 is emanating from an exacerbated cellular stress response consisting of enhanced R-loop formation, increased DNA DSBs, and extensive replication stress, collectively culminating in the activation of caspase-3 to initiate cell death.

The importance of PARP1 activity in counteracting the cellular stress response instigated by XRN2 deficiency and promoting cell survival is becoming evident. Both lung and breast cancer cells depleted in XRN2 using previously validated siRNAs show sensitivity to clinically relevant PARP inhibitors, Rucaparib and Olaparib, over a range of concentrations (Figure 1). These data are consistent with the sensitivity of XRN2-deficient non-cancer fibroblast cells toward Talazoparib [20] and LN229 glioblastoma cells toward Niraparib [26]. Also, the deficiency of another transcription termination factor, Kub5-Hera/RPRD1B that interacts with XRN2 and promotes its recruitment to termination sites [20,44,47], also shows similar sensitivity to PARP inhibition [33]. Our findings highlight the crosstalk between RNA and DNA metabolism mediated by XRN2 and strengthen the underlined translational implications.

The enhanced PARPi sensitivity of XRN2-deficient cells is driven by an aggravated cellular stress response that involves significantly higher R-loop formation (Figure 2), when compared to the individual deficiency or inhibition of XRN2 and PARP1. We utilized the S9.6 antibody to evaluate R-loops in the current study. However, the non-specific binding of the S9.6 antibody presents several limitations in evaluating the R-loops [30,48,49,50]. Notably, to ensure that the S9.6 foci are specific to the RNA–DNA hybrids of R-loops (Figure 2), we employed several measures in the present study including focusing on nuclear R-loop foci, excluding S9.6 signal overlapping with nucleolin and demonstrating RNase H-sensitivity of nuclear S9.6 signal in our experimental and control samples used in the immunofluorescence analyses. Also, we utilized dot blot analyses as another independent method to evaluate R-loops. Moreover, elevated R-loops in XRN2-deficient cells have been reported by others using S9.6-based ChIP- and DRIP-seq approaches [26,32]. Additionally, we and others have shown previously that XRN2-deficient cells accumulate R-loops during active transcription that can be removed by the overexpression of RNase H [25,26]. Essentially, XRN2 deficiency promoting R-loop formation presented here is consistent with the findings from other studies [25,26,32]. Recently, PARP1 was reported to interact with RNA–DNA hybrids and implicated in the R-loop biology [36]. PARP1 inhibition promoting R-loop formation observed here is consistent with recent reports demonstrating enhanced RNase H-sensitive RNA–DNA hybrid formation after siRNA-mediated depletion or pharmacological inhibition of PARP1 [36,51]. Also, we recently defined the XRN2 interactome and showed that it physically interacts with PARP1 [20]. Collectively, the emerging interplay of XRN2 and PARP1 in R-loop metabolism is intriguing and further investigation is ongoing in our laboratory.

R-loops have emerged as a potent source of genomic instability, especially DSB formation [52,53]. Previously, we showed that both PCNA-positive and PCNA-negative XRN2-deficient cells display significantly higher levels of DSB formation compared to control cells, indicating that XRN2 deficiency instigates DNA damage in both replicating and non-replicating cells [25]. These observations were further validated through subsequent studies [20,26,32]. Consistent with these studies, here, we observed that XRN2 deficiency alone in lung and breast cancer cells instigates elevated DSB formation (Figure 3 and Figure 4). It is conceivable that, in XRN2-deficient cells, PARP1 plays an important role in coordinating the resolution of elevated R-loops and/or sensing consequent DNA damage. Thus, compromised PARP1 function in XRN2-deficient cells could lead to dire consequences. This notion is supported by the data presented here, which indicate that XRN2 deficiency combined with PARPi renders cells to accumulate R-loops (Figure 2), consequently creating elevated DSBs (Figure 3 and Figure 4) and ultimately activating caspase-3 to initiate cell death (Figure 7). Moreover, similar to XRN2 deficiency, PARP1 inhibition results in elevated DSB formation in both replicating and non-replicating cells [51]. We previously showed that XRN2 deficiency not only elevates basal levels of DSBs but also compromises the repair of these breaks due to the loss of classical non-homologous end joining (cNHEJ) [25]. Recently, another study reported that compromised cNHEJ in XRN2-deficient cells is likely arising from abrogated Ku70 binding at the sites of DSBs [26]. The authors also described that XRN2 loss causes an extended association of EXO1 to chromatin, resulting in extensive DNA end resection and inhibition of the homologous recombination (HR) pathway of the DSB repair [26]. Enhanced PAR (poly(ADP-ribose)) formation in cancer cells presented here (Figure 4) is a direct consequence of increased R-loop formation and subsequent DNA damage instigated by XRN2 depletion. This notion is supported by the following observations: (i) XRN2 deficiency leads to RNase H-sensitive R-loops (Figure 2); (ii) XRN2-deficient cells show R-loop-dependent DSB formation [25,26]; (iii) PARP1′s physical interaction with R-loops results in its catalytic activation [51]. Taken together, these findings further support the PARPi sensitivity of XRN2-deficient cancer cells presented in this study.

Earlier, we reported that XRN2-deficient cells show increased replication stress, including elevated levels of phosphorylated Chk1^Ser317^ and phosphorylated RPA32^Ser4/8^ [25] and others showed that depletion of XRN2 does not alter cell cycle distribution or cell growth [26,54]. Moreover, PARP1 inhibition alone has also been shown to increase replication stress [55] and is known to cause G2/M arrest. Individual depletion of XRN2 or inhibition of PARP1 creating replication stress that we observed here (Figure 6) is consistent with our previous study [25]. A potential caveat could be the defective Okazaki fragment processing in XRN2-deficient cells that could also lead to replication stress and PARP1 activation independent of the R-loop formation [56]. This possibility is further complicated by the S9.6 antibody’s capacity to bind RNA–DNA hybrids longer than six base pairs and potentially recognize unprocessed Okazaki fragments. However, the direct role of XRN2 in DNA replication has not been explored yet; currently, evidence of XRN2 deficiency promoting Okazaki fragment processing defects is lacking. Regardless of this possibility, mechanistically the augmented replication stress in XRN2-deficient cells treated with PARP1 inhibitors (Figure 6) is attributed, at least partially, to elevated R-loop formation, and it is imperative to their synthetic lethal relationship since XRN2 deficiency or PARP1 inhibition alone causes R-loop formation and R-loops are a potent source of replication stress [52,53].

## 5. Conclusions

In conclusion, at the molecular level, XRN2 deficiency combined with PARP inhibition in cancer cells exacerbates R-loop formation, elevates the generation of DSBs and replication stress, and consequently drives the activation of caspase-3 to initiate cell death. Our findings emphasize the potential translational significance of targeting XRN2 cancer vulnerabilities via PARPi given that PARP1 inhibition impairs its compensatory role in mitigating cellular stress created by XRN2 loss.

## Data Availability

All data are available in the main text or the supplementary material.

## Supplementary Materials

The following supporting information can be downloaded at: https://www.mdpi.com/article/10.3390/cancers16030595/s1, Raw images of Western blots are included in the supplementary material.

## Abbreviations

R-loops	RNA–DNA hybrids with a displaced single-stranded DNA
DSBs	DNA double-strand breaks
HR	Homologous recombination
NHEJ	Non-homologous end joining
PAR	Poly(ADP-ribose)
PARPi	PARP inhibitors
