# Supplementary material for: Molecular Basis of XRN2-Deficient Cancer Cell Sensitivity to Poly(ADP-ribose) Polymerase Inhibition
Source: Cancers (Basel). 2024 Jan 30;16(3):595. doi: 10.3390/cancers16030595 (PMC10854503; doi:10.3390/cancers16030595)

Supplementary Materials

Molecular basis of XRN2-deficient cancer cell sensitivity to PARP inhibition

Talysa Viera, Quinn Abfalterer, Alyssa Neal, Richard Trujillo, Praveen L. Patidar*

Department of Chemistry, New Mexico Institute of Mining and Technology, Socorro, NM, 87801, USA

*To whom correspondence should be addressed: Praveen L. Patidar, Department of Chemistry, New Mexico Institute of Mining and Technology, 801 Leroy Pl., Socorro, NM, 87801, USA; Email: Praveen.Patidar@nmt.edu; Phone: +1 575 835 5168; Fax: +1 575 835 5364

Figure S1 Figure 1C Supplementary

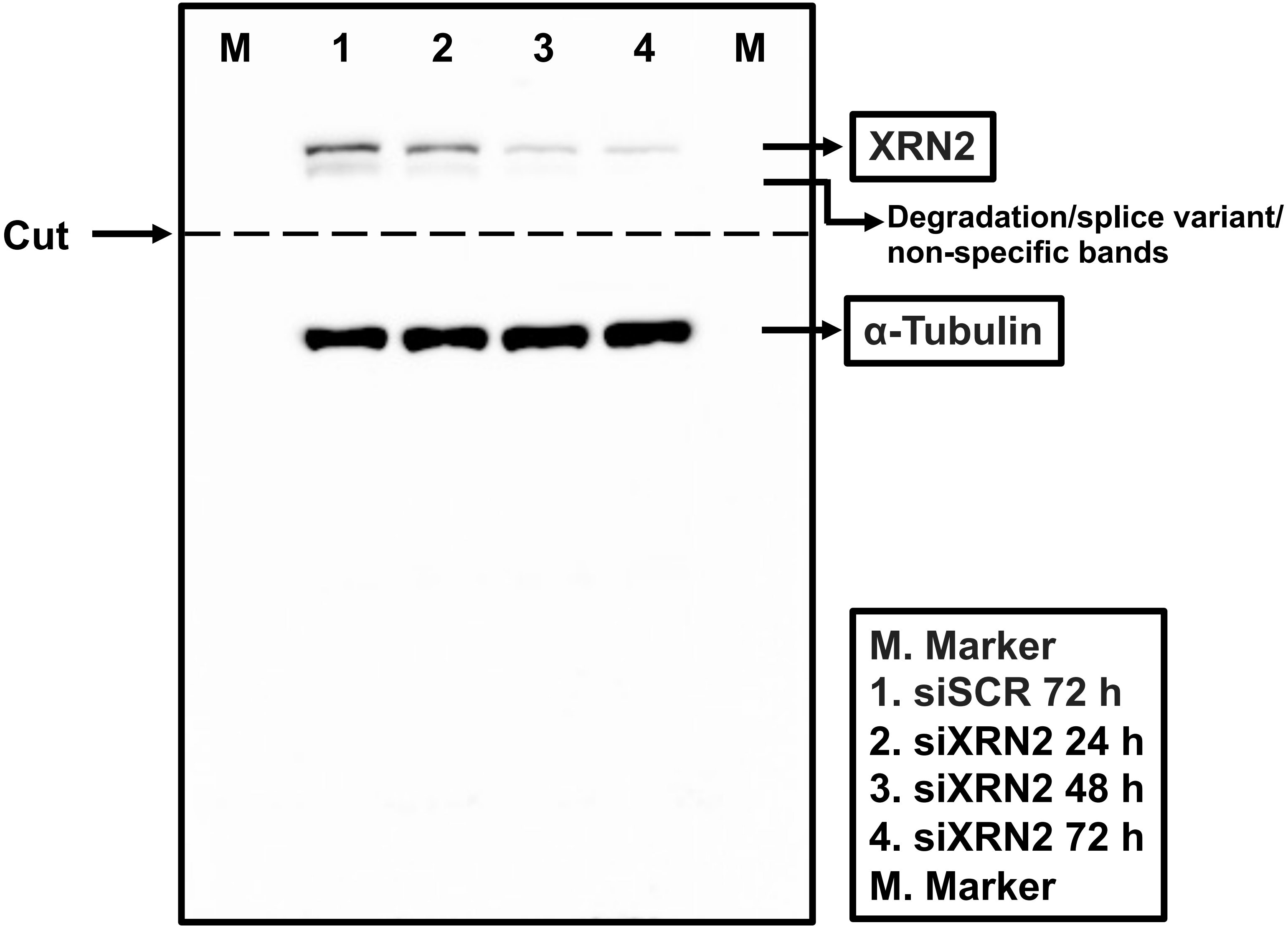

Figure S1 Figure 1F Supplementary

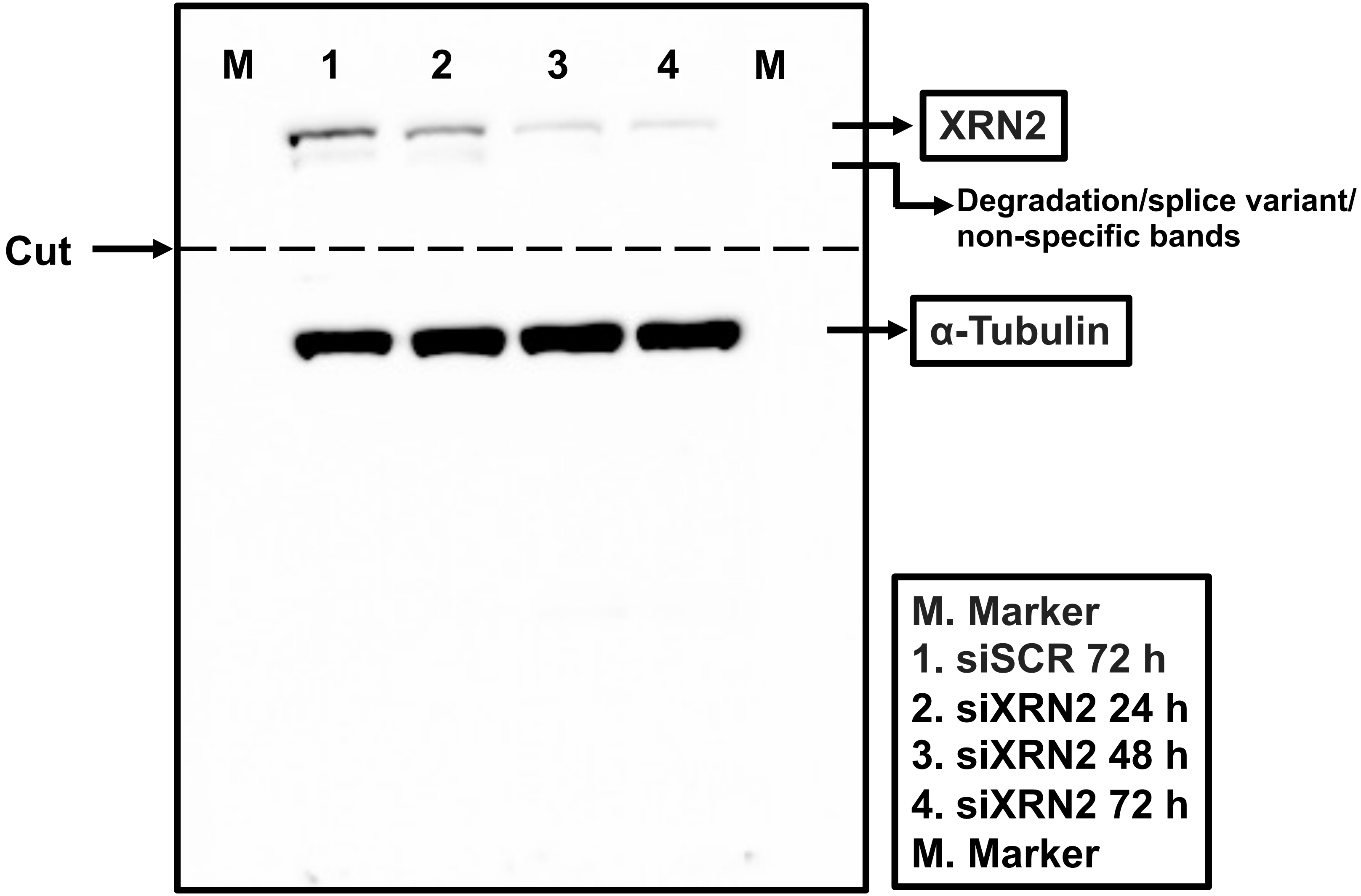

Figure S1 Figure 1I Supplementary

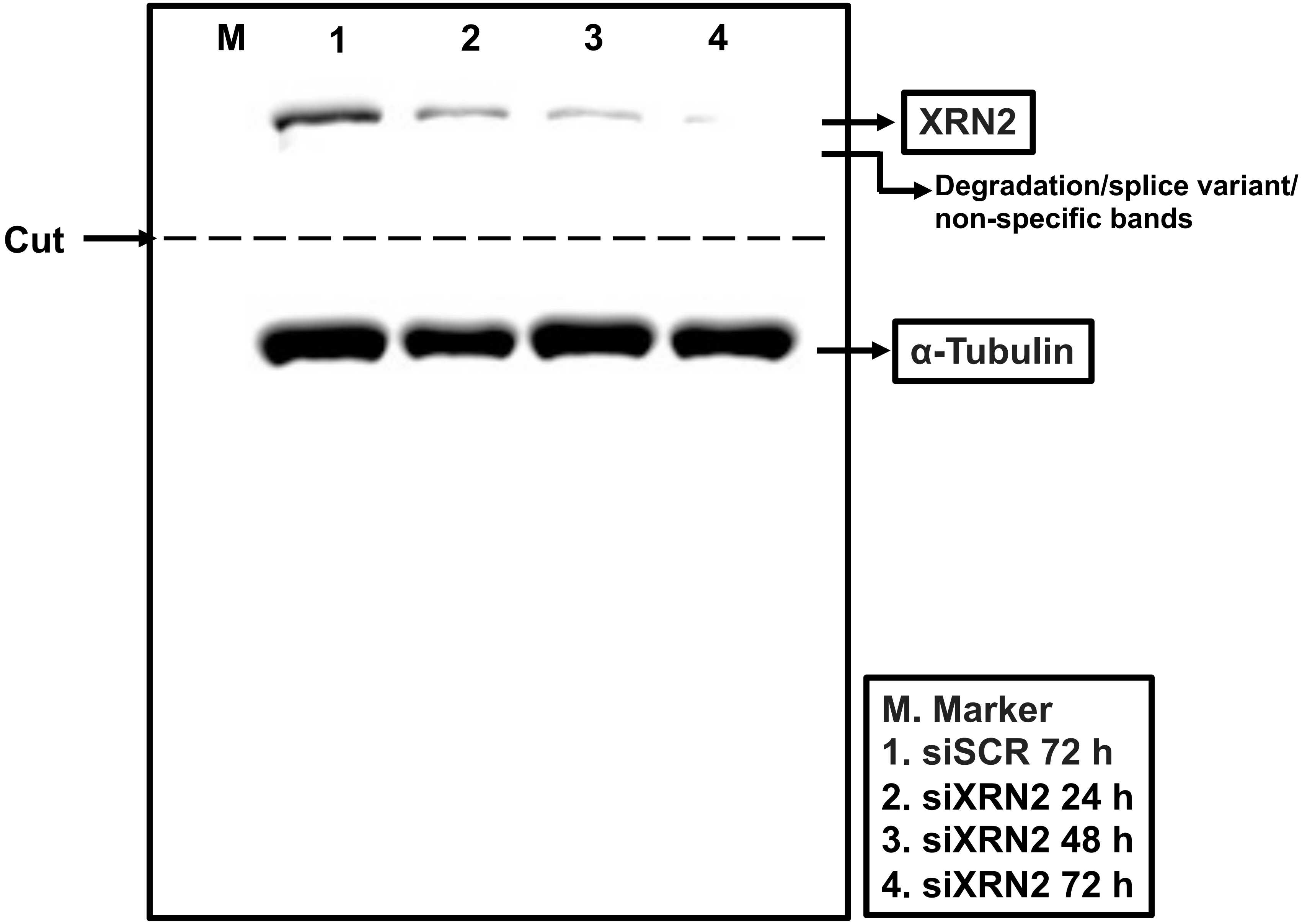

Figure S1 Figure 1L Supplementary

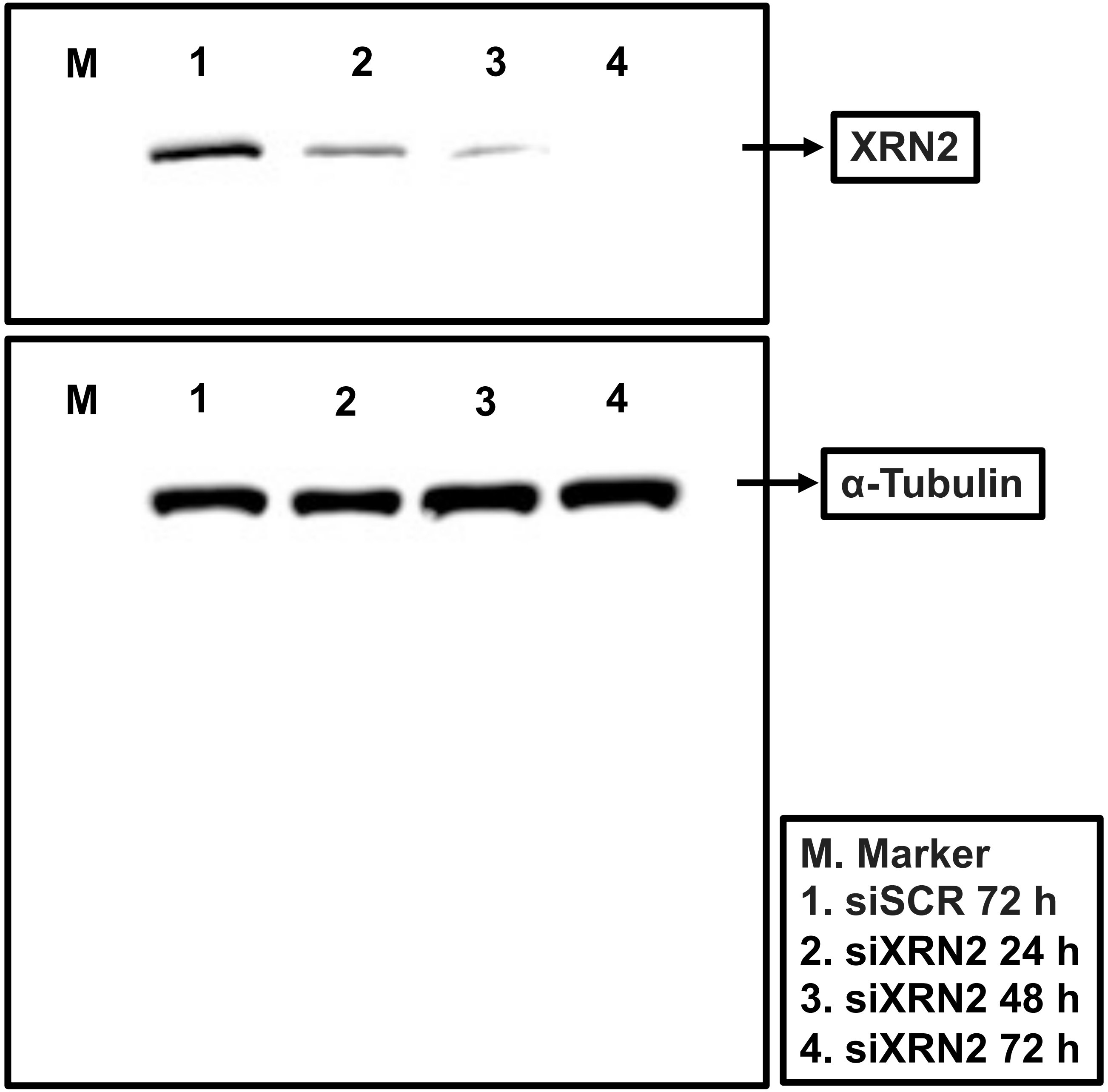

Figure S2 Figure 2D Supplementary

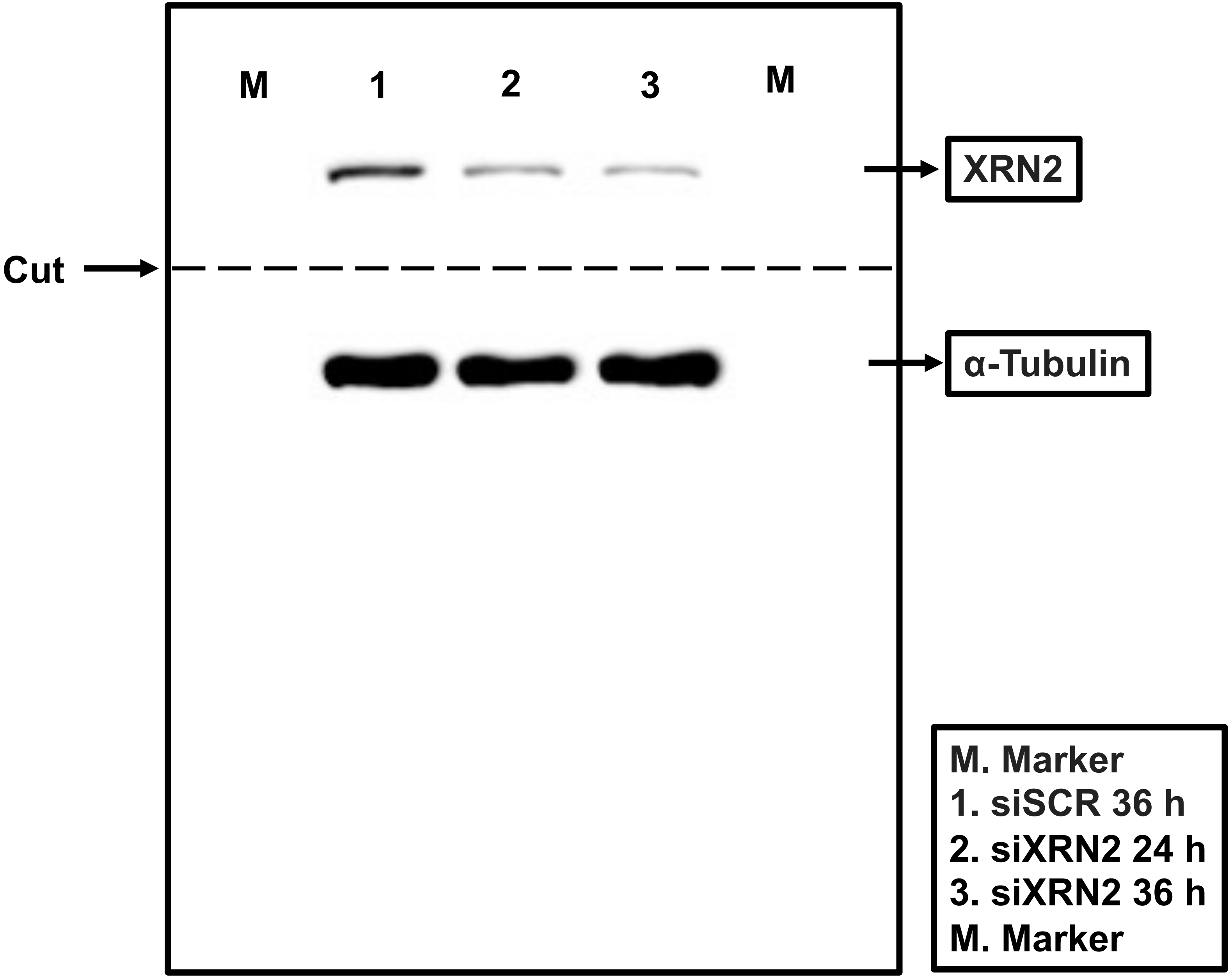

Figure S2 Figure 2G Supplementary

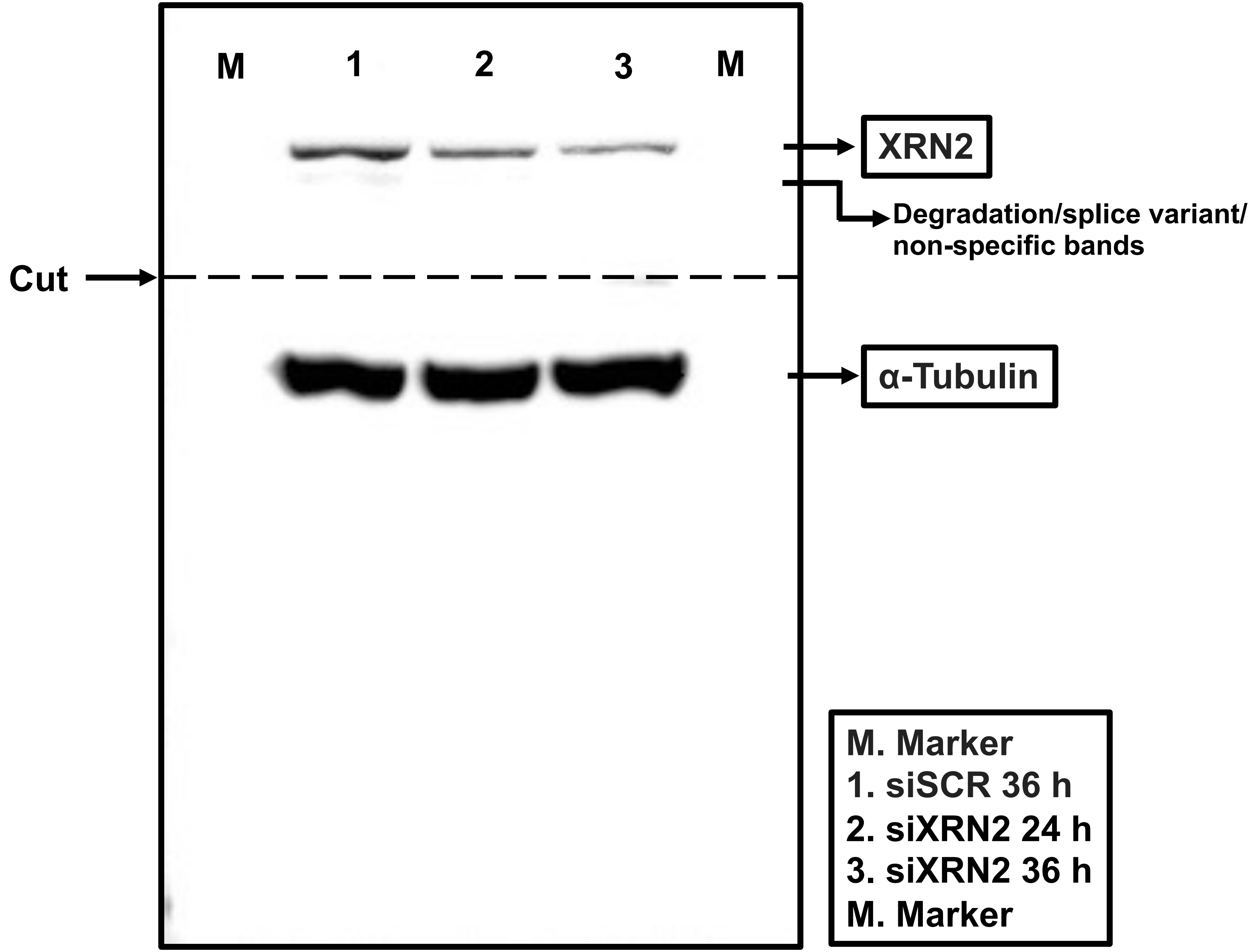

Figure S3 Figure 3C Supplementary

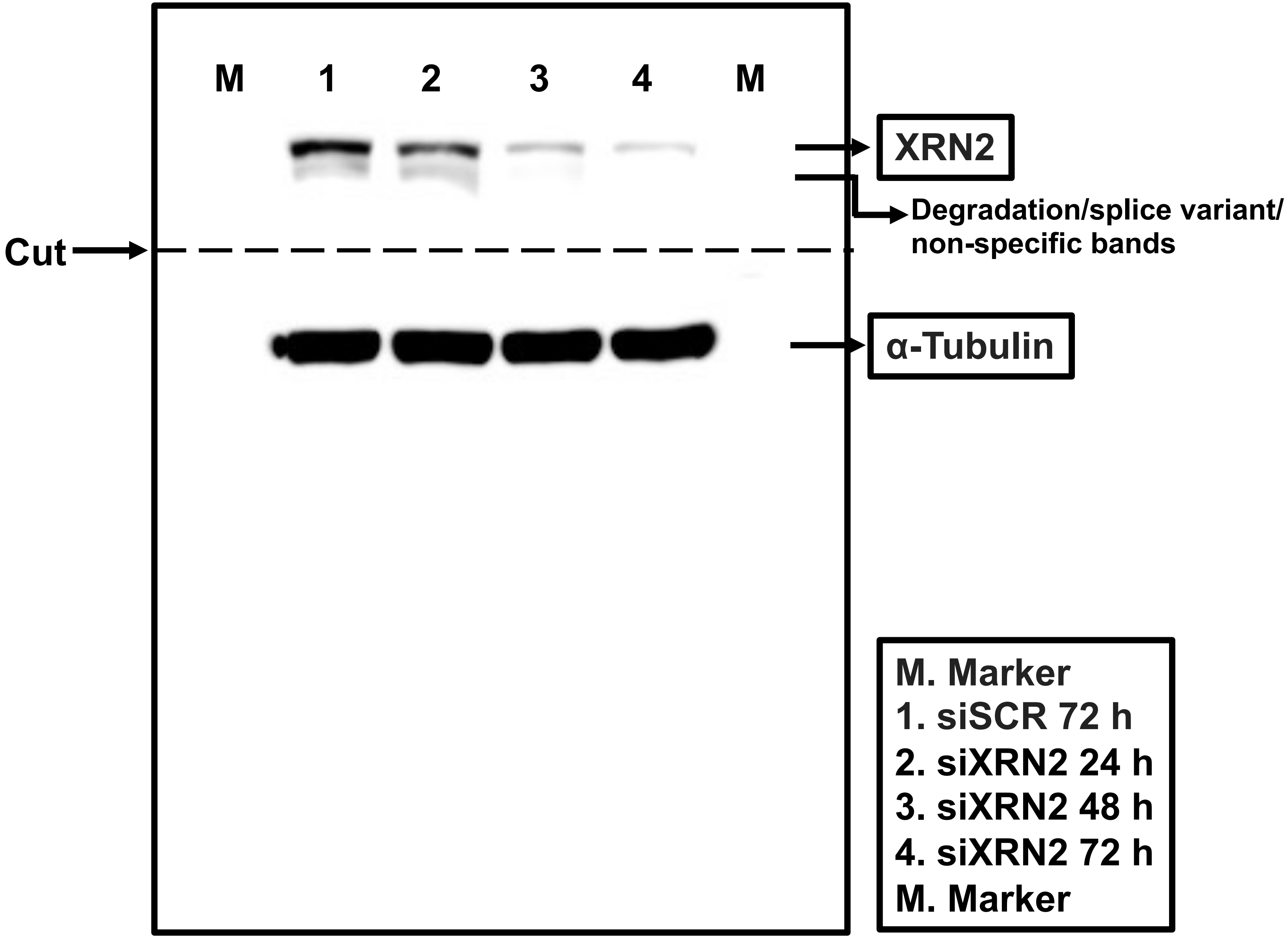

Figure S3 Figure 3F Supplementary

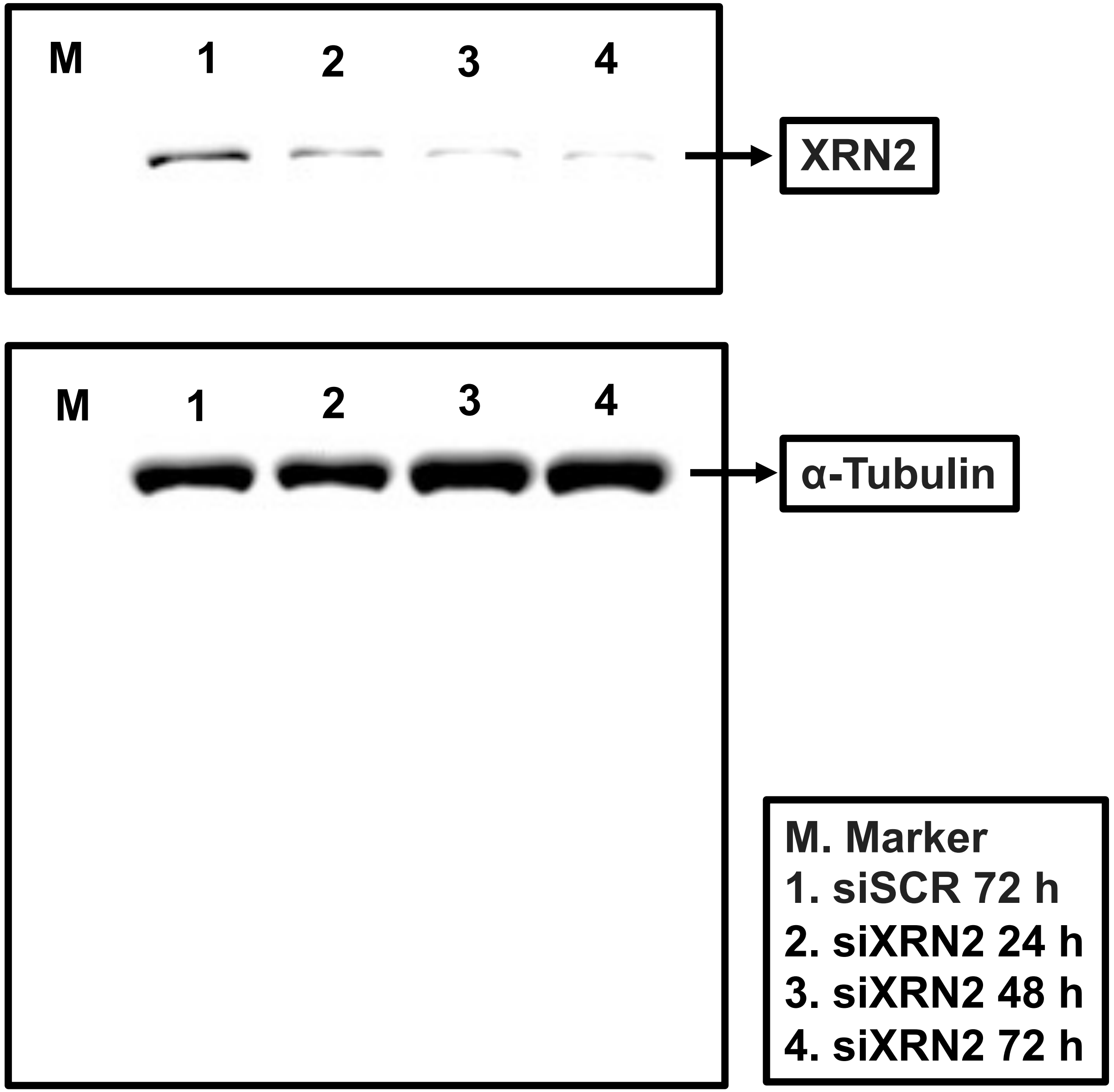

M. Marker
1. siSCR 72 h
2. siXRN2 24 h
3. siXRN2 48 h
4. siXRN2 72 h

Figure S4 Figure 4D Supplementary

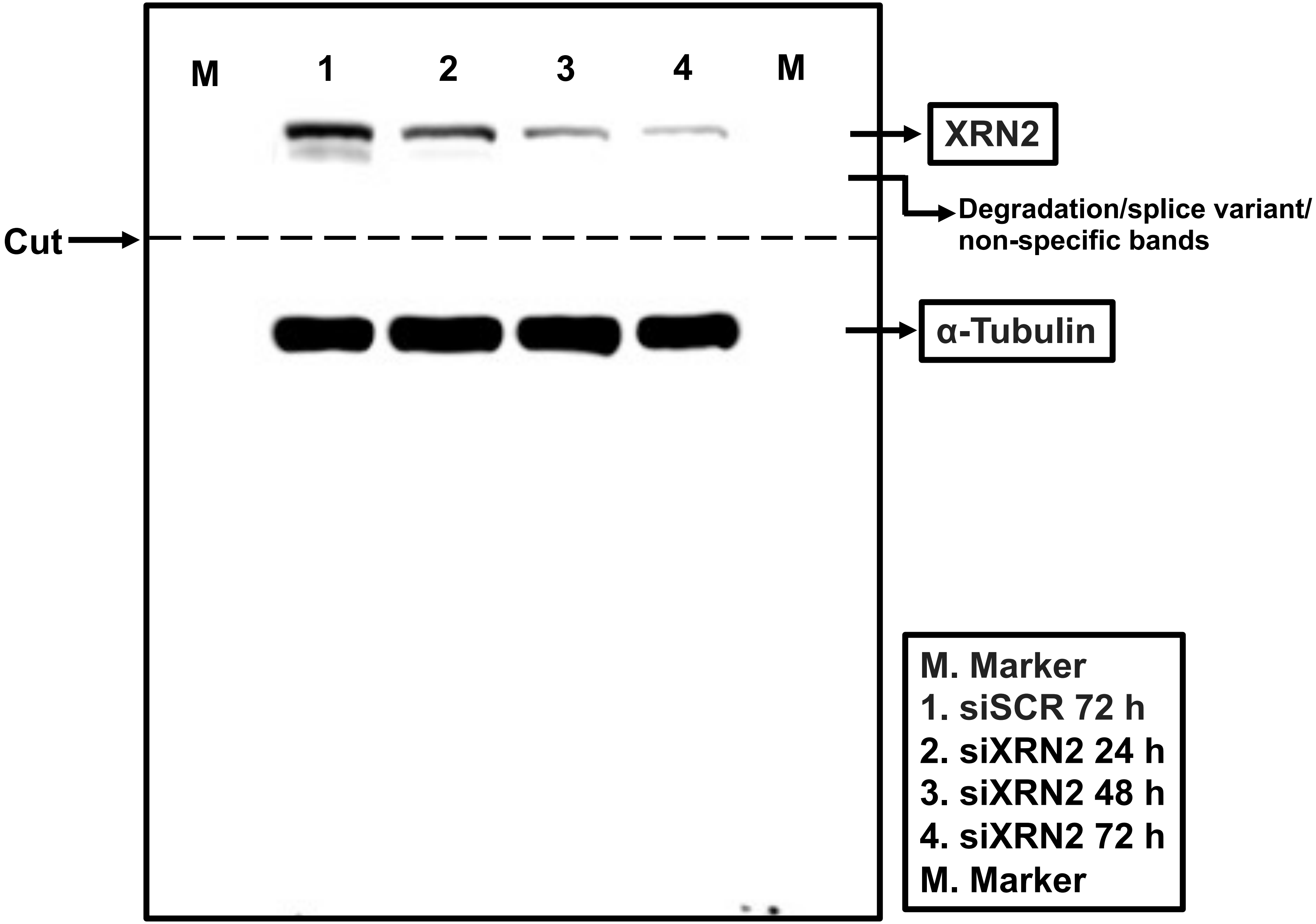

Figure S4 Figure 4H Supplementary

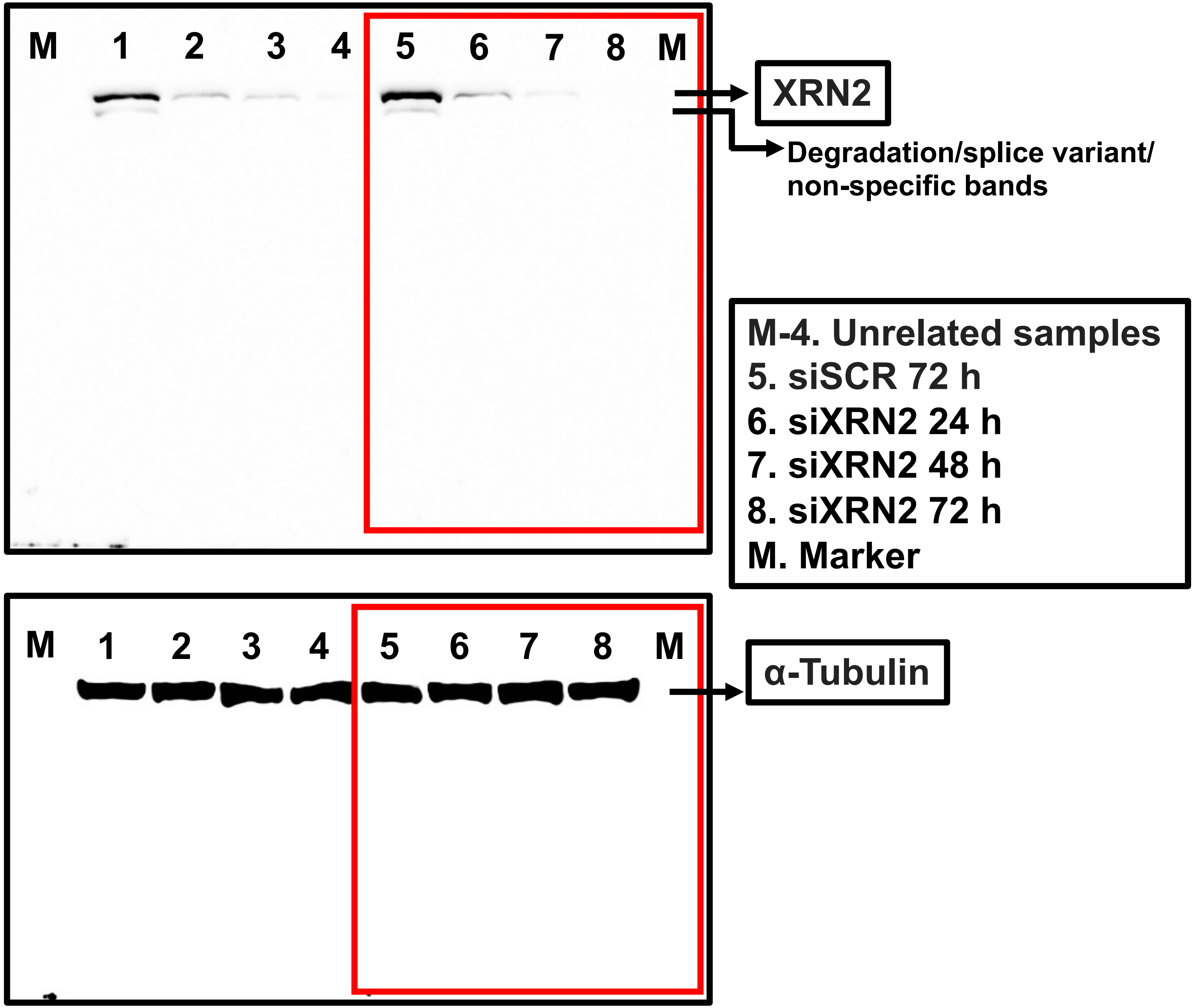

Figure S5 Figure 5B Supplementary

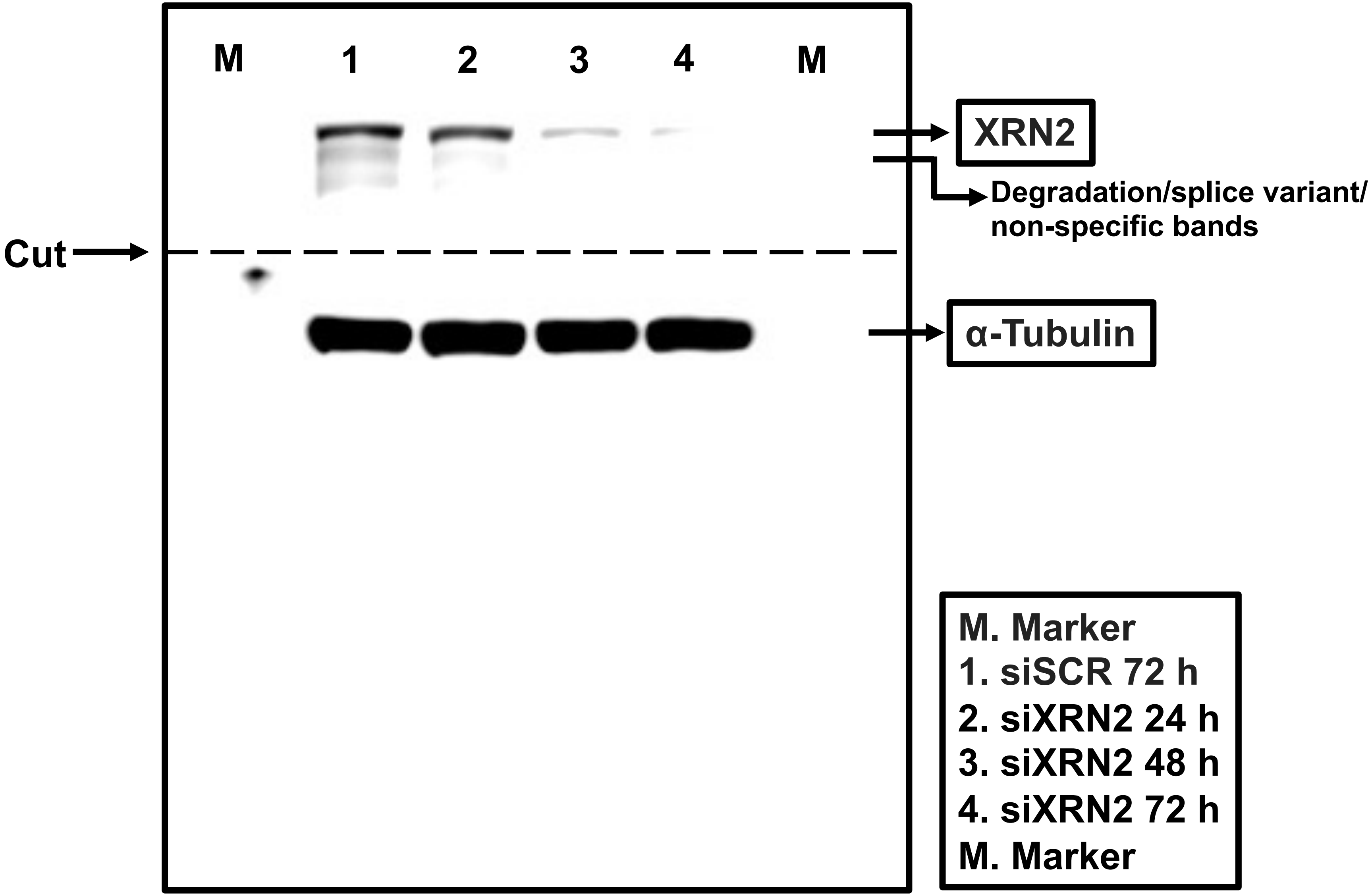

Figure S5 Figure 5D Supplementary

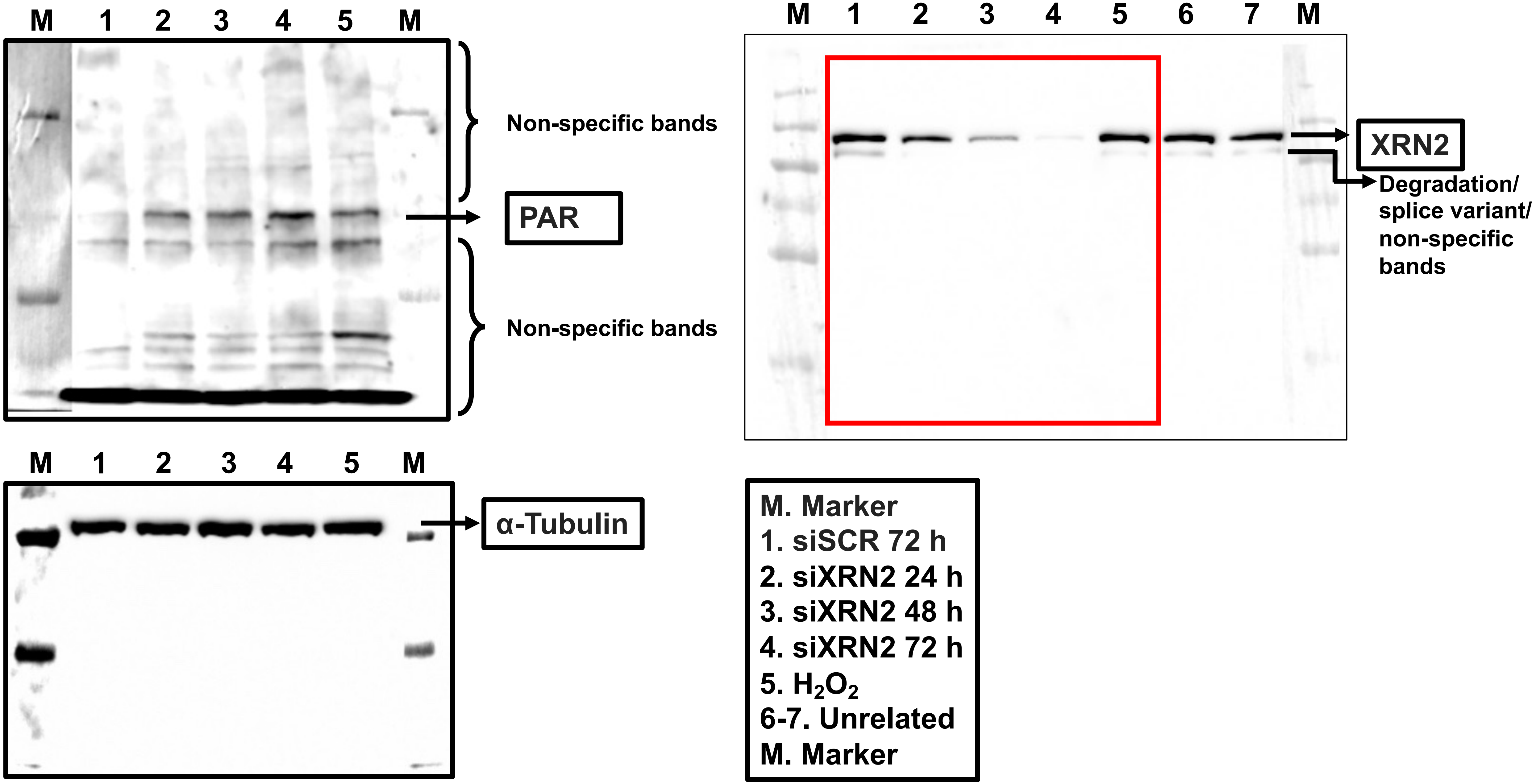

M. Marker
1. siSCR 72 h
2. siXRN2 24 h
3. siXRN2 48 h
4. siXRN2 72 h
5. H_2O_2
6-7. Unrelated
M. Marker

Figure S5 Figure 5E Supplementary

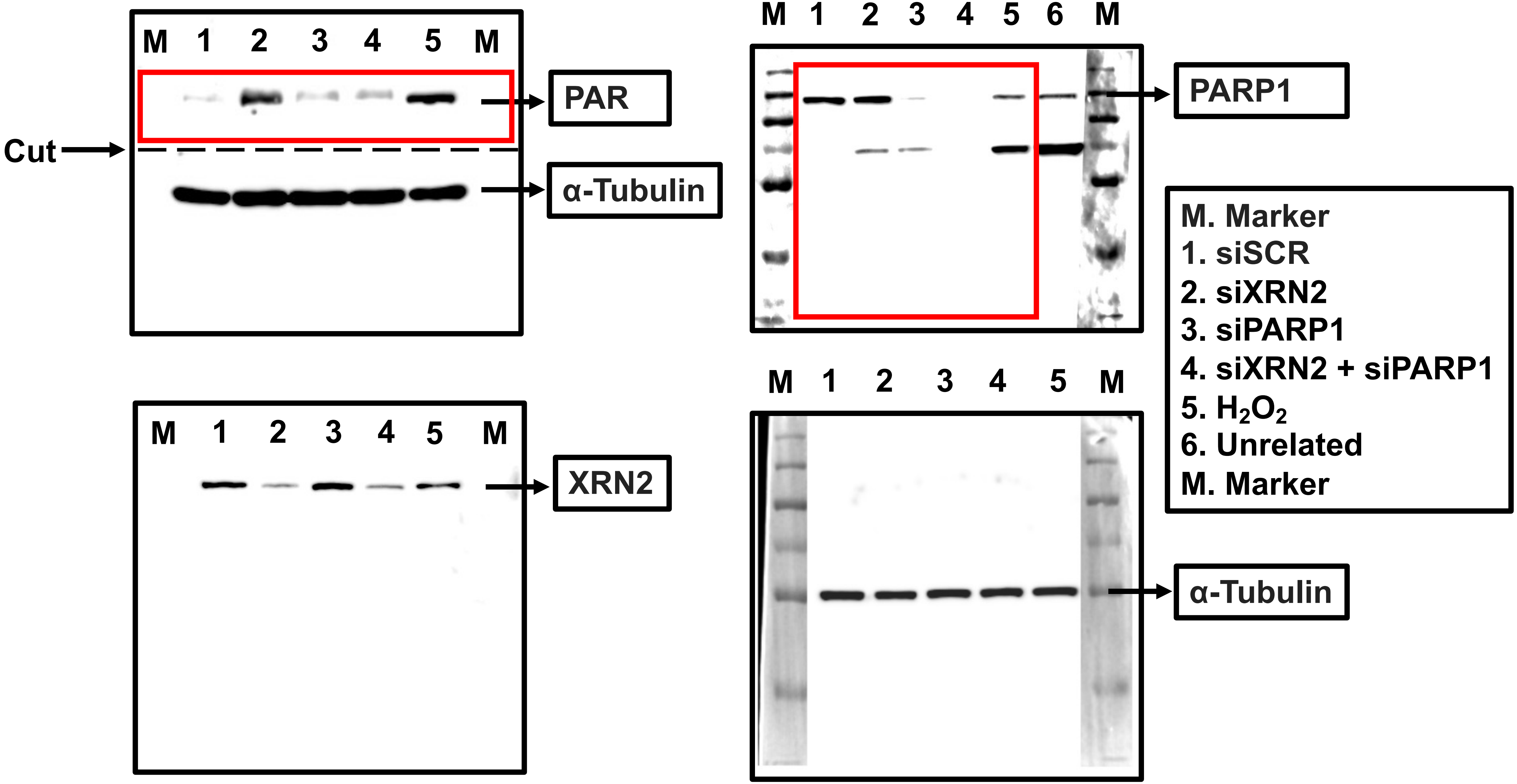

Figure S5 Figure 5I Supplementary

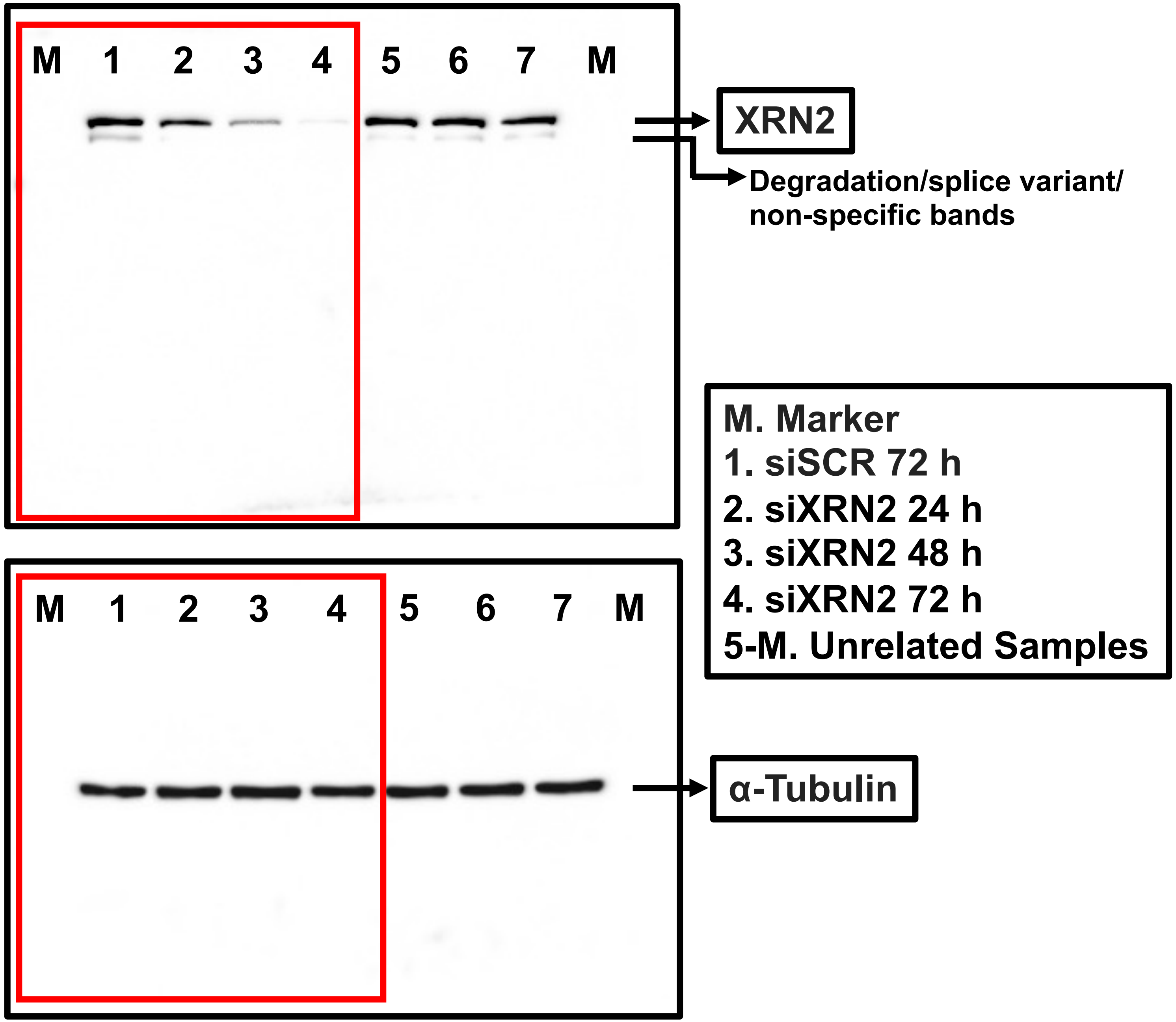

Figure S6 Figure 6D Supplementary

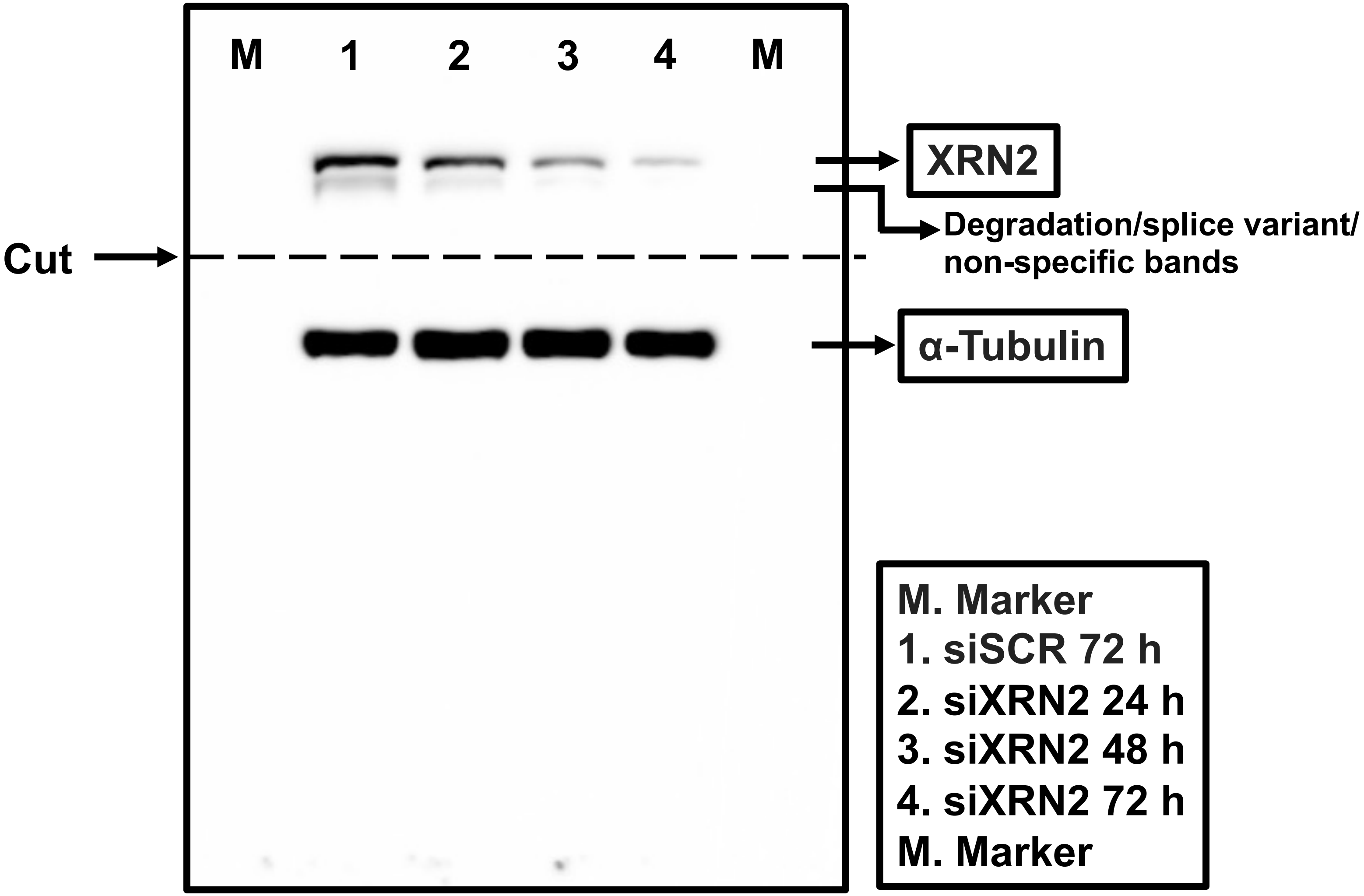

Figure S6 Figure 6H Supplementary

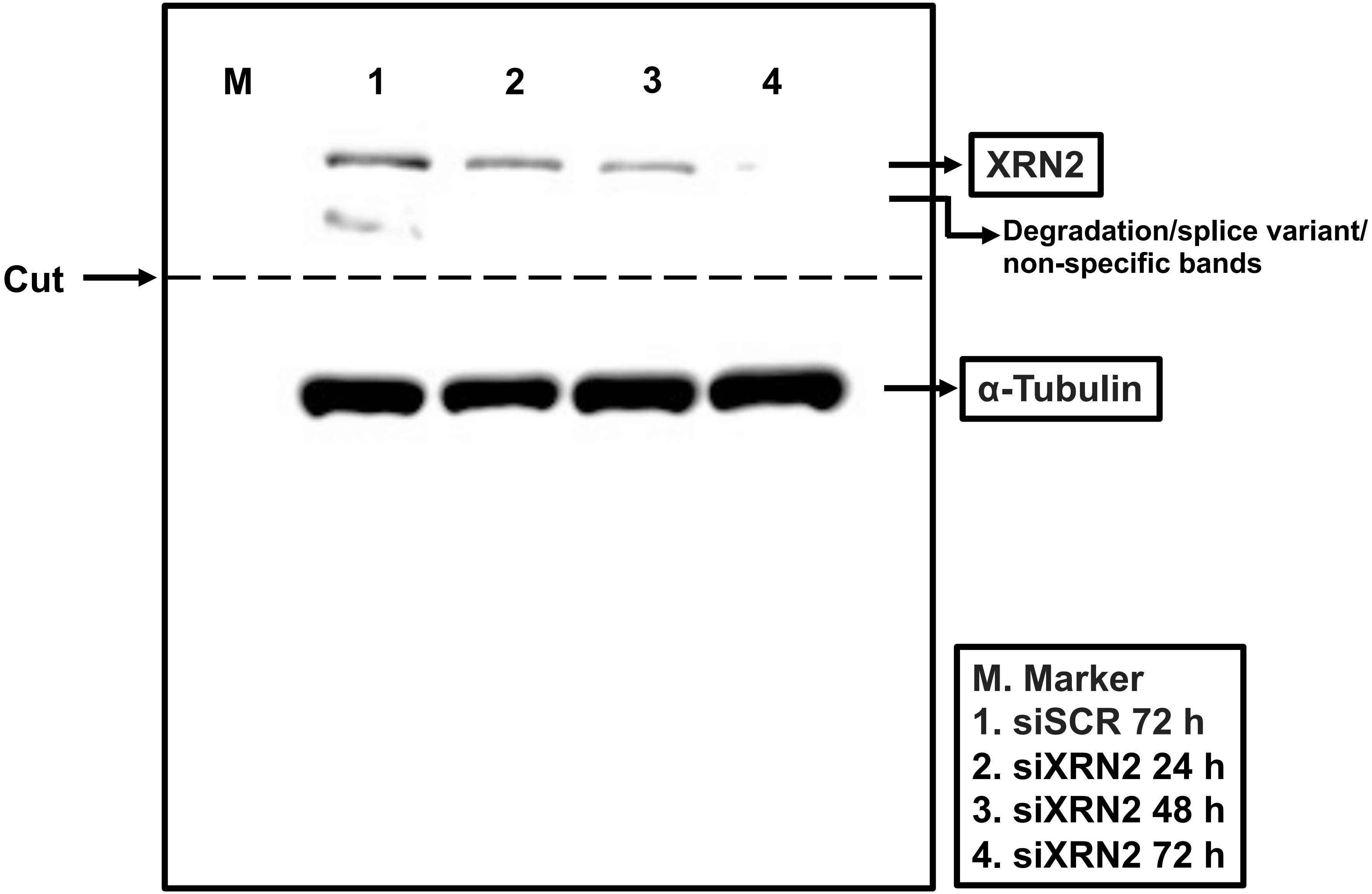

Figure S7 Figure 7C Supplementary

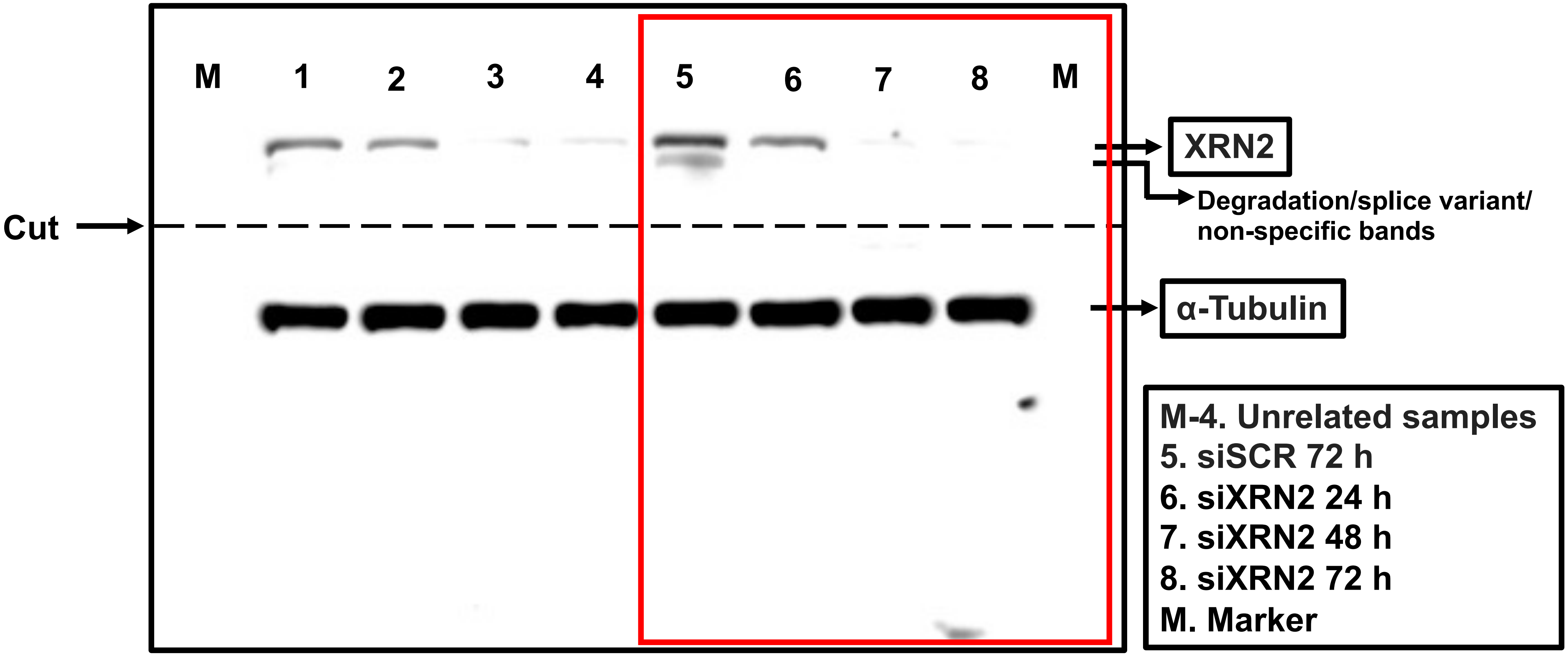

Figure S7 Figure 7F Supplementary

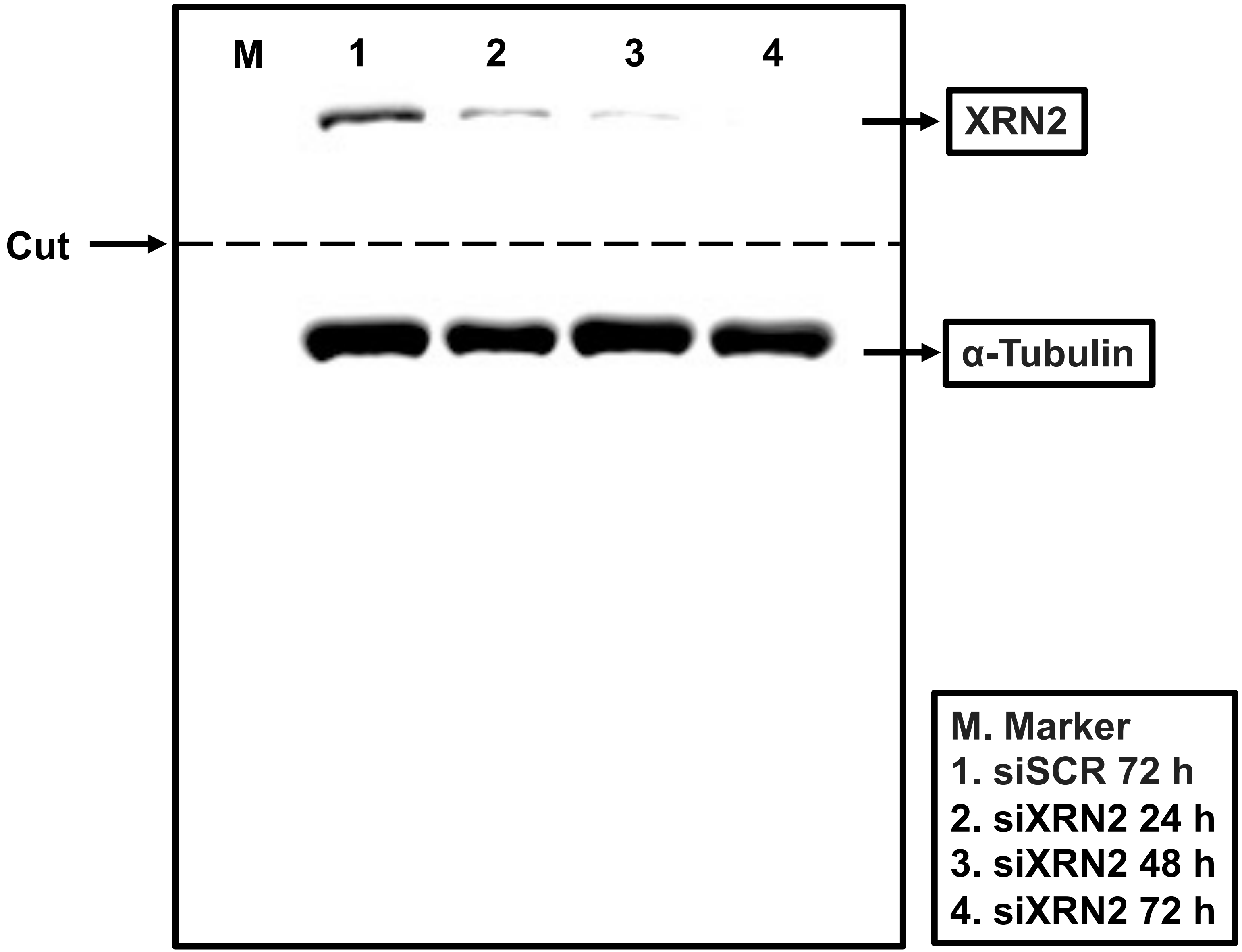

Supplement: Supplementary file 1 [file cancers-16-00595-s001.zip › cancers-2812405-supplementary.pdf]
